# A self-enforcing HOXA11/Stat3 feedback loop promotes stemness properties and peritoneal metastasis in gastric cancer cells

**DOI:** 10.7150/thno.36277

**Published:** 2019-10-14

**Authors:** Chao Wang, Min Shi, Jun Ji, Qu Cai, Jinling Jiang, Huan Zhang, Zhenggang Zhu, Jun Zhang

**Affiliations:** 1 Department of Oncology, Ruijin Hospital, Shanghai Jiao Tong University School of Medicine, No. 197 Ruijin er Road, Shanghai, 200025, China.; 2 Shanghai Institute of Digestive Surgery, Ruijin Hospital, Shanghai Jiao Tong University School of Medicine, No. 197 Ruijin er Road, Shanghai, 200025, China.; 3 Department of Radiology, Ruijin Hospital, Shanghai Jiao Tong University School of Medicine, No. 197 Ruijin er Road, Shanghai, 200025, China.

**Keywords:** peritoneal metastasis, gastric cancer, HOXA11, Stat3, BBI608

## Abstract

**Rationale:** Peritoneal metastasis is one of the most common and life-threatening metastases in gastric cancer patients. The disseminated gastric cancer cells forming peritoneal metastasis exhibit a variety of characteristics that contrast with those of adjacent epithelial cell of gastric mucosa and even primary gastric cancer cells. We hypothesized that the gene expression profiles of peritoneal foci could reveal the identities of genes that might function as metastatic activator.

**Methods:** In this study, we show, using *in vitro, in vivo, in silico* and gastric cancer tissues studies in humans and mice, that Homoebox A11 (HOXA11) potently promote peritoneal metastasis of gastric cancer cells.

**Results:** Its mechanism of action involves alternation of cancer stemness and subsequently enhancement of the adhesion, migration and invasion and anti-apoptosis. This is achieved, mainly, through formation of a positive feedback loop between HOXA11 and Stat3, which is involved in the stimulation of Stat3 signaling pathway.

**Conclusions:** These observations uncover a novel peritoneal metastatic activator and demonstrate the association between HOXA11, Stat3 and cancer stemness of gastric cancer cells, thereby revealing a previously undescribed mechanism of peritoneal metastasis.

## Introduction

Most patients with gastric cancer do not succumb to their primary tumor but instead to peritoneal metastasis that become apparent after the primary lesion has been removed. Surprisingly, not all the disseminated gastric cancer cells can form the peritoneal metastasis successfully, this indicates that a specific subset of gastric cancer cells, and/or microenvironment in the peritoneum, might already have a manner of selecting the specific or appropriate gastric cancer cells to colonize, and as such, could provide us with insights into preventing and/or curing peritoneal metastasis of gastric cancer cells. For gastric cancer cells to contribute to peritoneal metastasis, they must detach from the primary site, survive in the peritoneal cavity, and then adhere to the peritoneal mesothelium and colonize the subperitoneal tissues 1. This led us to hypothesize about factors that induce or maintain disseminated gastric cancer cells to form peritoneal metastasis. As such, the gene expression profiles of gastric cancer cells in peritoneal foci could serve as a source of metastasis-related genes, enabling us to uncover previously undescribed dependencies and vulnerabilities of peritoneal metastasis. In one of our prior studies, the variant expression of genes in the foci of primary gastric cancer, peritoneal metastases and adjacent chronic gastritis tissue in one case of gastric cancer patient were measured using RNA-seq technology 2.

Here we analyze the gene expression profiles of aforementioned RNA-sequencing examination to identify an oncogene, Homeobox A11 (HOXA11), which was a transcription factor and has been reported to play contradictory roles in lung adenocarcinoma 3, lung squamous cancer 4, renal cell carcinoma 5, urothelial bladder cancer 6 and glioblastoma 7. Gene ontology analysis has shown that HOXA11 may exert its function through the focal adhesion pathway 3, Furthermore, during the human female reproductive tract development, expression of HOXA11 was enhanced in human uterine mesenchyme 8, and HOXA11 was found to be crucial in skeletal stem cells to maintain the skeletal development and self-renew throughout the life of the animal^9^. In our experiment, HOXA11 can be found in nucleus and positively affect the peritoneal metastasis ability of gastric cancer cells* in vivo*. The mechanism of action of this oncogene involves, mainly, alteration of gastric cancer stemness, which are accompanied by anoikis/apoptosis-resistant, metastases and adhesion.

## Materials and methods

### Cell Line and cell culture

Gastric cancer cell lines (KATO III, NCI-N87, SNU-16, AGS and SNU-16) and HEK 293T were purchased from American Type Culture Collection (ATCC, Manassas, VA, USA), other gastric cancer cell lines and GES-1 cells were obtained from the Chinese Academy of Science. Human peritoneal mesothelial cells (HPMCs) were obtained from patients and validated by immunofluorescence. All cell lines have been authenticated by short tandem repeat (STR) profiling and tested to exclude mycoplasma contamination before experiments. All cells were cultured in DMEM-medium supplemented with 10% fetal bovine serum (FBS), 100 U/ml Penicillin G and 100 μg/ml streptomycin sulfate, and maintained at a 37 °C incubator with a 5% CO_2_ atmosphere and less than 3 months after resuscitation.

### Clinical patient tissue microarray

The microarrays which contained primary gastric cancer and paired adjacent normal paraffin-embedded (FFPE) specimens (n=190) were purchased from Shanghai outdo biotech company. The company provided us with a written informed consent that the research was conducted in accordance with recognized ethical guidelines. This experiment was approved by the Ethics Committee of Shanghai Jiaotong University, School of Medicine Affiliated Ruijin Hospital and was conducted in accordance with ethical principles of the World Medical Association Declaration of Helsinki as well.

The gastric cancer specimens were divided into two groups via X-tile software 10: the first 114 were termed as training cohort and the remaining 76 as validation cohort. Conventional clinic pathological factors comprising age, gender, gastric cancer stage (AJCC) and tumor volume were recorded and listed in Table S1. Overall survival (OS) time was defined as the interval between surgery and time of death. For patients that passed away, the intervals were censored at the date of death, moreover for patients that lived, they were censored at the final follow-up.

Gene expression data of RNA-Seq in peritumor and primary tumor of gastric cancer patients were downloaded from TCGA and GEO database. The relationship between HOXA11 expression and the prognosis of gastric cancer patients was analyzed online (http://kmplot.com/analysis/index.php?p=service&cancer=gastric), which contained GSE14210, GSE15459, GSE22377, GSE29272, GSE51105 and GSE62254 for the GEO datasets.

### Immunohistochemistry staining and evaluation

Immunohistochemistry staining of tissue microarrays and paraffin sections of mice' peritoneal tumor tissues which was carried out on a 4 μm-thick slices were confirmed to be tumor by hematoxylin and eosin (HE) staining, then followed by En Vision two-step procedure of Dako REAL^TM^ Envision^TM^ Detection System (Dako, Agilent Technologies, Ca, USA). When antigen retrieval was performed, the slices were incubated with the primary antibodies overnight at 4 °C, and the next day, incubated with the HRP labeled secondary antibodies at 37°C for 30 minutes and finally all slides were visualized with diaminobenzidine. The antibodies were listed in Table S4.

HOXA11 protein was localized in cell nucleus and stained as brownish granules. The expression status of HOXA11 was calculated by the mean of density (IOD/Area) via Image pro plus software. We defined 0.31 as the cutoff value of HOXA11 for high and low expression according to the analysis of X-tile software based on their relationship with OS in the training cohort, and then the cutoff value was further validated in the validation cohort.

### Plasmids transfection and virus transduction

For plasmid transfections, cells were transfected using Lipofectamine 2000 (Cat. 11668-019; invitrogen), according to the manufacturer's instructions and selected with 5 μg/ml puromycin (Cat. Ant-pr-5; Invivogen). The shRNA sequences for HOXA11 and CD44s were designed and synthesized by GenePharma, and the sequences are shown in Table S6. The above cells were then used for further experiments. HOXA11 and CD44s deletion were confirmed by western blot.

For virus transduction, cells were transducted with polybrene, according to the manufacturer's instructions and selected with 5 μg/ml puromycin (Cat. Ant-pr-5; Invivogen). The HOXA11 gene was constructed in lentiviral vector (pWPXL). Overexpression of HOXA11 were confirmed by western blot, qRT-PCR and Immunofluorescence. The stable cell lines which HOXA11 had been overexpressed were then used for further experiments.

### Immunofluorescence

Gastric cancer cells were fixed in 4% formaldehyde for 30 minutes, and then permeabilizated with 0.1% Triton X-100 for 30 minutes. After that cell were treated with primary antibodies overnight at 4 °C atmosphere, followed by species-specific secondary antibodies conjugated with Cy3 and DAPI which was then used to stain the nucleus the next day at room temperature. The results were observed, recorded and analysed using Carl Zeiss microscope, ZEN software (ZEISS Company) and Image J software. The antibodies are shown in Table S4.

### Co-immunoprecipitation and Western blotting

Co-immunoprecipitation (Co-IP) were carried out as the protocol of Pierce Co-Immunoprecipitation Kit (Cat. 26149, Thermo scientific). Briefly, NCI-N87-HOXA11 or SGC-7901-HOXA11 lysates were prepared in IP Lysis/Wash Buffer. 1 mg lysate was pre-cleared with 80 μl control agarose resin slurry at 4 °C for 1 hours with gentle end-over-end mixing, and then the supernatant was added into the spin columns containing the corresponding antibodies-coupled resin and incubated with rocking overnight at 4 °C. The beads were washed with 60 μl Elution buffer and boiled for 5 minutes, and then followed with western blot detection.

The nuclear and cytoplasmic protein of Stat3 were extracted as per the protocol of nuclear and cytoplasmic protein extraction kit (#P0027, Beyotime). The protein samples were separated by SDS-PAGE (12.5%) and transferred onto polyvinylidene fluoride (PVDF) membranes using the wet blotting system (BIO-RAD). The membranes were blocked with 5% BSA in TBS-T, incubated for corresponding antibodies overnight at 4 °C, and subsequently incubated with species-specific secondary antibodies for 2 hours at room temperature. The infrared imaging system (LI-COR Biosciences) and ECl substrate solution (Cat. P10300, NCM Biotech) were used to visualize the immunoreactive proteins. The band intensity was densitometrically evaluated using Image J software. The antibodies were listed in Table S4.

### RNA isolation and quantitative real-time RT-PCR analysis

Total RNA was extracted using TRIzol reagent method (Invitrogen). Complementary DNA (cDNA) was obtained via reverse transcription of equal amount of RNA following protocols and subsequently mixed with primers, SYBR Green PCR MIX (Applied Biosystems) and germ-free water. The reaction products were run on ABI Prism 7900HT sequence detection system (Applied Biosystems), and serial cDNA dilutions were used to generate GAPDH intensity reference standards according to the comparative Ct method^11^. The primer sequences of all genes were listed in Table S5.

### Chromatin immunoprecipitation assay

Chromation immunoprecipitation (ChIP) assays were essential performed as per the protocol of Chromatin Immunoprecipitation Kit (Cat. 17-371, Millipore). Briefly, NCI-N87-HOXA11 and SGC-7901-HOXA11 cells were fixed in 1% formaldehyde before being quenched with 1× glycine respectively. Cells were washed twice with PBS and followed by lysis and sonication to produce DNA fragments of between 200-1000 bp. Chromatin was pre-cleared and ChIP was performed using chromatin equivalent to 1×10^6^ cells. Chromatin was incubated with antibody overnight at 4 °C and then incubated with protein G agarose for 1 hour. Immunoprecipitated DNA was eluted from the agarose and subsequently reverse crosslinked by 5 M NaCl, RNase A, 0.5 M EDTA, 1 M Tris-HCL and Proteinase K. DNA was isolated by phenol/chloroform extraction and ethanol precipitation. The primers sequences of HOXA11 segments are listed in Table S5.

### Luciferase reports assay

The human wild type and mutant HOXA11 promoter region (27185217-27187216) were cloned into the pGL3-basic vector. NCI-N87 and SGC-7901 cells were plated in 24-well and co-transfected with the wild-type, mutant HOXA11 promoter luciferase reporter plasmids and pRL-TK vector with pcDNA3.1-Stat3 or pcDNA3.1. For the groups treated with BBI608, cells were treated with certain concentration of BBI608 for 24 h. Finally, Cells were collected 48 h after transfection and luciferase activity was analyzed with the Dual-Luciferase Reporter Assay System (Promega). Renilla luciferase was used to normalize reporter luciferase activity, which rescaled to vector control signals equal to unit 1. Experiments were repeated in triplicated.

### Bisulfite genomic DNA sequencing

Genomic DNA was isolated from peritumor tissues and primary tumor tissues of Gastric cancer patients using the Genomic DNA purification kit (Promega), which was followed by treatment with sodium bisulfite (QIAGEN #59824). The specific PCR primers were designed to amplify the HOXA11 promoter bisulfite-modified regions (+27185410 - +27185609): 5'- GGGGAYGAGAGTTGAGTTTTTAT-3' (forward); 5'- CACCTCAAAAAAAACAACAAATC-3' (reverse).

### Tumorsphere formation

NCI-N87-HOXA11, SGC-7901-HOXA11, MGC-803-shHOXA11 #1, MGC-803-shHOXA11 #2 cells and their negative control cells were, respectively, trypsinized into single cell suspension and seeded in an ultra-low attachment surface 24-well plate (Cat. 3473, Corning) at a density of 2000 cells/well. Cells were maintained in PromoCell 3D tumorsphere medium XF (Cat. C-28070, Promo Cell) and cultured at 37 °C for nine days. To assess self-renewal capabilities, tumorsphere were harvested and trypsinized into single cell suspension. Cells were cultured in a brand-new ultra-low attachment surface 24-well plate for a further nine days prior to scoring. Tumorspheres > 50 μm were scored using a graticle. For drug treatment, cells were incubated with 3 or 6.5 μm BBI608 (Cat. 83280-65-3, Selleck) for 24 hours.

### Isolation of anoikis-resistant cells

NCI-N87-HOXA11, SGC-7901-HOXA11, MGC-803-shHOXA11 #1, MGC-803-shHOXA11 #2 cells and their negative control cells were, respectively, trypsinized into single cell suspension and seeded in poly-HEMA coated 24 well plate (Cat. CBA-080, Cell Biolabs, Inc) at a density of 1×10^5^ cells/well and cultured in suspension for 72 hours at 37 °C. Anoikis-resistant cells were collected and trypsinized to form a single cell suspension for anoikis analysis.

### Flow cytometry analysis of apoptosis and anoikis

3×10^5^ cells were plated in each well of the 6-well plates and cultured for 72 hours, and subsequently, cells were collected and stained with 7-AAD (Cat. 51-68981E, BD Biosciences Corporation) and APC-Annexin V (Cat. 550474, BD Biosciences Corporation) as well as the above anoikis-resistant cell suspension have been done according to the same process. And subsequently a flow cytometric analysis was performed using FACS (Becton-Dickinson). For drug treatment, cells were incubated with 3 or 6.5 μM BBI608 (#83280-65-3, Selleck) for 24 hours.

### Wound healing and Transwell assay

NCI-N87-HOXA11, SGC-7901-HOXA11, MGC-803-shHOXA11 #1, MGC-803-shHOXA11 #2 cells and their negative control cells were, respectively, trypsinized into single cell suspension and seeded into 6-well plates at a density of more than 90%. Wounds were scratched with 20 μl sterile pipette tips and suspended cells were removed with PBS buffer washing, followed by adding serum-free medium for cell culture. Micrographs were taken every 24 hours. All experiments were performed in triplicate.

For migration assay, NCI-N87-HOXA11, SGC-7901-HOXA11, MGC-803-shHOXA11 #1, MGC-803-shHOXA11 #2, NCI-N87-HOXA11/shCD44s, SGC-7901-HOXA11/shCD44s cells and their negative control cells were, respectively, trypsinized into single cell suspension and placed in Boyden chambers (1×10^5^ cells/ chamber in serum-free DMEM medium and 8 μm for 24-well plate, Millipore), that were placed in DMEM medium containing 10% FBS and incubated at 37 °C for 24 hours.

For invasion assay, Boyden chambers were previously coated with Matrigel overnight, after the aforementioned cells had been incubated for 48 hours, boyden chambers were washed in PBS and cells were fixed in 1% paraformaldehyde and stained with 1% crystal violet. Non-migrated cells inside the chambers were removed and washed in PBS. Pictures of 5 different fields were captured. The ability of migration or invasion were calculated by the mean of number of migrated or invaded cells observed per field respectively. For inhibition with chemical drugs, cells were treated with BBI608 (Cat. 83280-65-3, Selleck; 3 or 6.5 μM) or CK636 (Cat. 442632-72-6, Selleck; 4 μM) during the time of migration or invasion assay.

### Cell-cell adhesion assay

NCI-N87-HOXA11, SGC-7901-HOXA11, MGC-803-shHOXA11 #1, MGC-803-shHOXA11 #2, NCI-N87-HOXA11/shCD44s, SGC-7901-HOXA11/shCD44s cells, their negative control cells as well as HPMCs were cultured in monolayer in 24-well plate for 48 hours (1×10^5^ cells/well). Gastric cancer cells were pre-treated with or without the addition of BBI608 (Cat. 83280-65-3, Selleck; 3 or 6.5 μM) for 24 hours and stained with calcein AM (1 μl per well) at 37 °C for 30 minutes. Afterwards, gastric cancer cells were trypsinized into single cell suspension and added to the wells cultured with HPMCs (5×10^4^ cells/well) and then incubated at 37 °C for 3 hours. The wells were subsequently washed with PBS for three times to remove the non-adherent cells. The remaining adherent gastric cancer cells were observed and captured using a fluorescence microscope. The difference was calculated by the numbers of gastric cancer adherent cells. All experiments were performed in triplicate.

### Peritoneal metastatic xenograft model

Male BALB/c nu/nu nude mice at the age of about 4 weeks (Institute of Zoology Chinese Academy of Sciences) were housed at a specific pathogen-free environment and randomly divided into 6 groups (5 mice in each group) (Research Center of Experimental Medicine, Shanghai Jiaotong University School of Medicine Affiliated Ruijin Hospital). MGC-803-shHOXA11 #1, MGC-803 shHOXA11 #2, NCI-N87-HOXA11, SGC-7901-HOXA11 cells and their negative control cells were lentivirally transduced firefly luciferase (FFLuc) fusion vector (Genepharma) and selected with 10 μg/ml puromycin (Cat. Ant-pr-5; Invivogen). Stable gastric cancer cells were trypsinized and re-suspended in 100 μl PBS which contained 3×10^5^ gastric cancer cells and injected into the abdomen of mice, respectively. For the groups of drug treatment, BBI608 (20 mg/kg, i.p, twice a week) were given at the fourth day after the injection of gastric cancer cells and last for 4 weeks. Before euthanasia of the mice, tumor mass and distribution in abdomen were assessed by bioluminescence imaging (BLI, the IVIS Imaging System, Caliper Life Sciences). The sample size calculation was performed using Spectrum living image software, and subsequently peritoneal metastases were checked by gross specimens and microscopy. All experiments adhered to the NIH Guide for the Care and Use of Laboratory Animals.

### Statistical analysis

The chi-square test or Fisher's exact test was used for categorical variable comparison. Overall survival of the patients in the group of variable HOXA11 expression was calculated with the Kaplan-Meier method and compared by the log-rank test. Univariate and multivariate survival analyses were tested using the Cox proportional hazards regression model. For the multivariate model, we used 0.05 as the cut-off P-value to select the analyzed factors from the univariate analysis data. A forward stepwise Cox regression model was applied to select independent prognostic factors. Independent sample *t*-test and one-way analysis of variance (ANOVA) were performed in quantitative data analysis. Data were shown as mean and standard error of the mean (SEM). The nomograms were carried out based on the results of multi-variate analysis and by using the package of rms in R version (http://www.r-project.org). The predictive accuracy of the nomogram was assessed by concordance index (C-index) and checked by calibration comparing nomogram-predicted *vs.* observed Kaplan-Meier estimates of survival probability. The prognostic prediction was more accurate when the C-index was larger, and in general, a C-index value larger than 0.75 was considered to represent relatively good discrimination. All statistical analyses were performed using SPSS 23.0 for windows (SPSS Inc.) and statistical programming language R for windows (cran.r-project.org). Two-tailed P-value less than 0.05 were considered as statistically significant.

## Results

### HOXA11 was high expressed in the peritoneal foci of gastric cancer and promoted peritoneal metastasis

**Figure 1:**
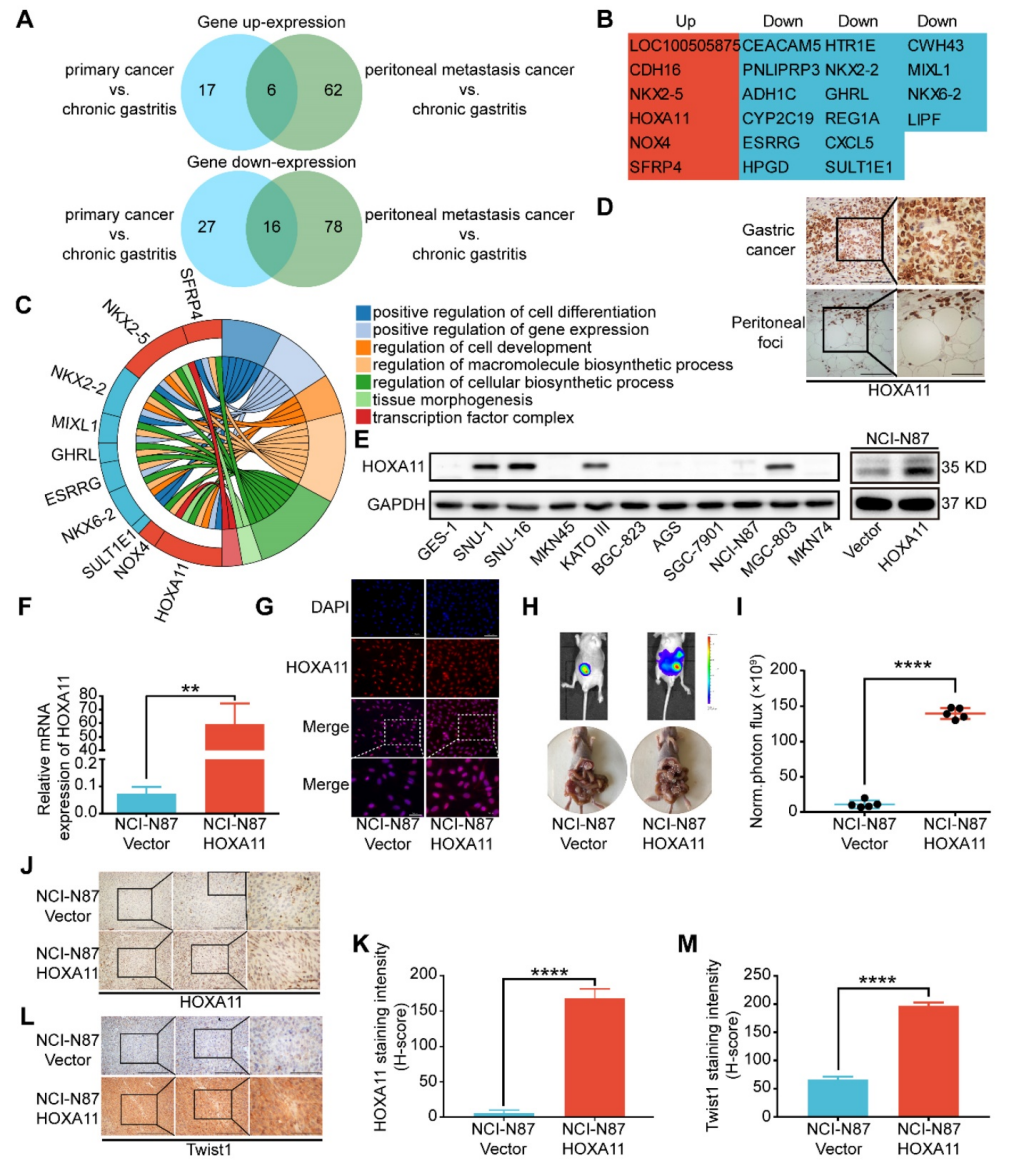
HOXA11 was high expressed in the peritoneal foci of gastric cancer and promoted peritoneal metastasis. (A) A venn diagram summarized the upregulated genes and downregulated genes in both primary gastric cancer and peritoneal foci when compared with the adjacent chronic gastritis tissues. (B) The list shown the genes' name which belong to the category of upregulated and downregulated genes, respectively. (C) Chordal graph shown the pathway analysis of shared upregulated and down-regulated genes in both primary gastric cancer and peritoneal foci by GO enrichment. (D) Immunohistochemistry assay show the expression of HOXA11 in both primary gastric cancer and peritoneal foci, the left scale bar, 200 μm, 200× magnification, the right scale bar, 100 μm, 400× magnification. (E) Left: western blot analysis of HOXA11 protein levels in 10 gastric cancer cells and normal gastric epithelial cell line GES-1, right: expression of HOXA11 of indicated cells were analyzed using western blot, and GAPDH was used as a loading control. Each experiment was performed in triplicate. (F) Expression of HOXA11 of indicated cells were analyzed using qRT-PCR. Results were shown as mean ± SEM of three independent experiments, each experiment was performed in triplicate. **, P<0.01 (Student *t* test). (G) Immunofluorescence staining for HOXA11 in NCI-N87-Vector and NCI-N87-HOXA11 cells are shown here (HOXA11, red; DAPI, blue). The scale bar, 100 μm, 200× magnification; 50 μm, 400× magnification. Each experiment was performed in triplicate. (H) Overexpression of HOXA11 promoted peritoneal metastasis of gastric cancer cells in BALB/c mice. Tumor in both groups are measured both in situ and after laparotomy. (I) Statistical analysis of the bioluminescence in peritoneal foci of both groups. Results were shown as mean ± SEM, ****, P<0.0001 (Student* t* test). (J & L) Immunohistochemistry assay shown the expression of HOXA11 and Twist1 in peritoneal foci derived from NCI-N87-Vector and NCI-N87-HOXA11 cell groups, respectively. The scale bar, from left to right, 400 μm, 100× magnification; 200 μm, 200× magnification; 100 μm, 400× magnification. (K & M) Statistical analysis of HOXA11 and Twist1 staining intensity (H-score) in both groups. Results were shown as mean ± SEM of three independent experiments, each experiment was performed in triplicate. ****, P<0.0001 (Student* t* test).

To discover the mechanism of peritoneal metastasis of gastric cancer, we re-analyzed the gene expression profiles of aforementioned RNA-sequencing examination 2. Comparing with adjacent chronic gastritis tissues, there are 22 shared genes which are variedly expressed in both of primary gastric cancer and peritoneal foci. Among them, 16 genes were down-regulated and 6 genes were up-regulated in both sites (Figure 1A & B). Gene ontology (GO) term enrichment analysis of the up-regulated and down-regulated genes were performed with the database for annotation, visualization and integrated discovery (DAVID) 12, 13. The results revealed that there were multiple genes involved in positive regulation of cell differentiation, positive regulation of gene expression, regulation of cell development, regulation of macromolecule biosynthetic process, regulation of cellular biosynthetic process, tissue morphogenesis and transcription factor complex (Figure 1C). HOXA11 was selected for further investigation since it fulfilled all the other criteria which have been chosen, such as: 1) The GEO database and TCGA database have shown that expression of HOXA11 is higher in gastric cancer rather than gastric tissue (Figure S4C-E). 2) Reconfirmation of RNA-sequencing data by immunohistochemical technology revealed strong expression of HOXA11 in both sites of primary gastric cancer and peritoneal foci (Figure 1D), 3) We further examined the expression of HOXA11 in gastric cancer cell lines and found that HOXA11 is highly expressed in SNU-16 cell which is derived from ascites, KATO III cell which is derived from pleural effusion, SNU-1 cell which is derived from a poorly differentiated primary carcinoma of the stomach as well as MGC-803, besides, there is almost no expression in GES-1 cells which belong to epithelial cells of gastric mucosa (Figure 1E left). 4) An extensive literature search found that no previous studies have discussed the function of HOXA11 in peritoneal metastasis of gastric cancer. 5) Elevated expression of HOXA11 was correlated with decreased gastric cancer patient survival rate in GEO database from the Kaplan-Meier plotter (www.Kmplot.com) (Figure S4G). Other ones in the set of shared genes did not meet all of the above criteria, which provide a strong rationale for thoroughly investigating function of HOXA11 in peritoneal metastasis of gastric cancer.

To determine whether HOXA11 could enhance the peritoneal metastatic ability of gastric cancer cells, we engineered NCI-N87 cell to overexpress HOXA11 (Figure 1E right, F & G), and injected them intraperitoneally into BALB/c mice, along with empty vector controls. The tumor burdens of HOXA11 group were obviously heavier than that of controls in the peritoneum of hosts (P<0.0001, Figure H & I). We extracted the peritoneal foci, processed them into paraffin embedded tissue sections and performed immunohistochemical analysis of the HOXA11 and metastatic marker Twist1, there were higher expression of Twist1 in HOXA11 group than the control ones (HOXA11: P<0.0001, Figure 1J & K; Twist1: P<0.0001, Figure 1L & M). The above findings indicated that HOXA11 might play a role in the peritoneal metastasis of gastric cancer.

### HOXA11 changed the morphology of gastric cancer cells and promoted gastric cancer cells motility, migration and invasion

**Figure 2:**
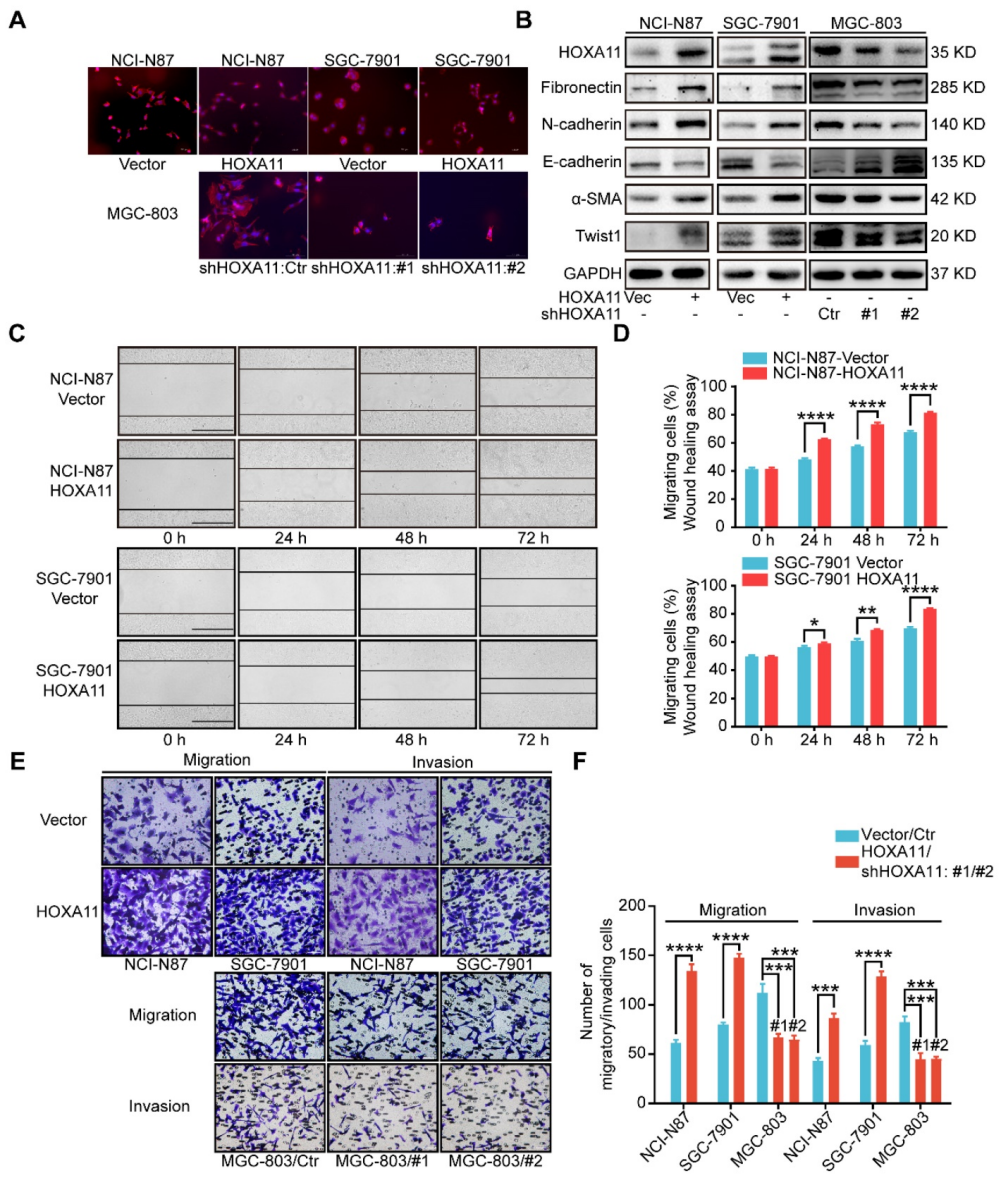
HOXA11 changed the morphology of gastric cancer cells and promoted gastric cancer cells motility, migration and invasion. (A) Fluorescence microscope images of NCI-N87 and SGC-7901 upon HOXA11 induction as well as MGC-803 cells upon HOXA11 knockdown. Rhodamine phalloidin was used to label the cytoskeletal F-actin (phalloidin, red; DAPI, blue). The scale bar, 100 μm, 200× magnification. Each experiment was performed in triplicate. (B) The protein expression of HOXA11, Fibronectin, N-cadherin, E-cadherin, α-SMA, and Twist1 in NCI-N87-Vector, NCI-N87-HOXA11, SGC-7901-Vector, SGC-7901-HOXA11, MGC-803-Control, MGC-803-shHOXA11 #1 and MGC-803-shHOXA11 #2 cells were analyzed using western blot with the indicated antibodies. GAPDH was used as the internal protein loading control. Each experiment was performed in triplicate. (C) Time-lapse phase-contrast images from scratch-wound assays performed on NCI-N87-Vector, NCI-N87-HOXA11 cells, SGC-7901-Vector and SGC-7901-HOXA11 cells. The scale bar, 1000 μm, 40× magnification. (D) Quantification demonstrated impaired directional migration with HOXA11 overexpression (data points = % of original wound area healed at the indicated time). Results were shown as mean ± SEM of three independent experiments, each experiment was performed in triplicate. *, P<0.05; **, P<0.01; ****, P<0.0001 (Student *t* test). (E) Representative images of cell migration and invasion assays. The scale bar, 120 μm, 200× magnification. (F) Statistical analysis of number of migratory/invading cells. Results were shown as mean ± SEM of three independent experiments, each experiment was performed in triplicate. ***, P<0.001; ****, P<0.0001 (Student* t* test and the analysis of variance test).

To perform proof-of-principle *in vitro* study, we constructed the HOXA11 over-expressed model in SGC-7901, MKN45 cells as well as employed short hairpin RNA (shRNA) to downregulate HOXA11 expression in MGC-803, SNU-16, even HEK 293T cells. Two HOXA11 shRNAs (268/#1 and 433/#2) were chosen for further study based on their HOXA11 knockdown efficiencies (93% and 80%) in HEK 293T cell (Figure 2B & Figure S1B-E).

Immunofluorescence assay confirmed that the gastric cancer cells, which HOXA11 was induced, have experienced the epithelial-mesenchymal transition, the cells showed less differentiated morphology, which was characterized by tight epithelial cobblestone islands (Figure 2A), decreased expression of markers of epithelial differentiation (E-cadherin) and increased mesenchymal markers (Fibronectin, N-cadherin, Twist1 and α-SMA) and vice versa (Figure 2B and Figure S1G). Immunofluorescence analysis confirmed that mesenchymal marker (N-cadherin) were upregulated in gastric cancer cells which HOXA11 was induced, by contrast, N-cadherin was downregulated in MGC-803 cell which HOXA11 was knocked-down (Figure S1I).

Wound healing assay, which examine cell motility in two-dimensional space, demonstrated that the interval in “HOXA11 groups” shrunk faster than that in “control ones” (NCI-N87: 24h: P<0.0001, 48h: P<0.0001, 72h: P<0.0001; SGC-7901: 24h: P<0.05, 48h: P<0.01, 72h: P<0.0001) (Figure 2 C & D). The transwell assay with or without matrigel, could mimic cell invasion or migration in three-dimensional space, the results shown that the number of cells passed through the membrane in “HOXA11 groups” are more than that in “control ones” (NCI-N87: migration: P<0.0001, invasion: P<0.001; SGC-7901: migration: P<0.0001, invasion: P<0.0001), in contrast, HOXA11 silencing could reduce the number of cells passed through the membrane (MGC-803: migration: #1: P<0.001 and #2: P<0.001, invasion: #1: P<0.001 and #2: P<0.001), which represent that HOXA11 could positively enhance the migration and invasion of gastric cancer cells (Figure 2E & F).

### HOXA11 enhanced gastric cancer cell stemness

**Figure 3:**
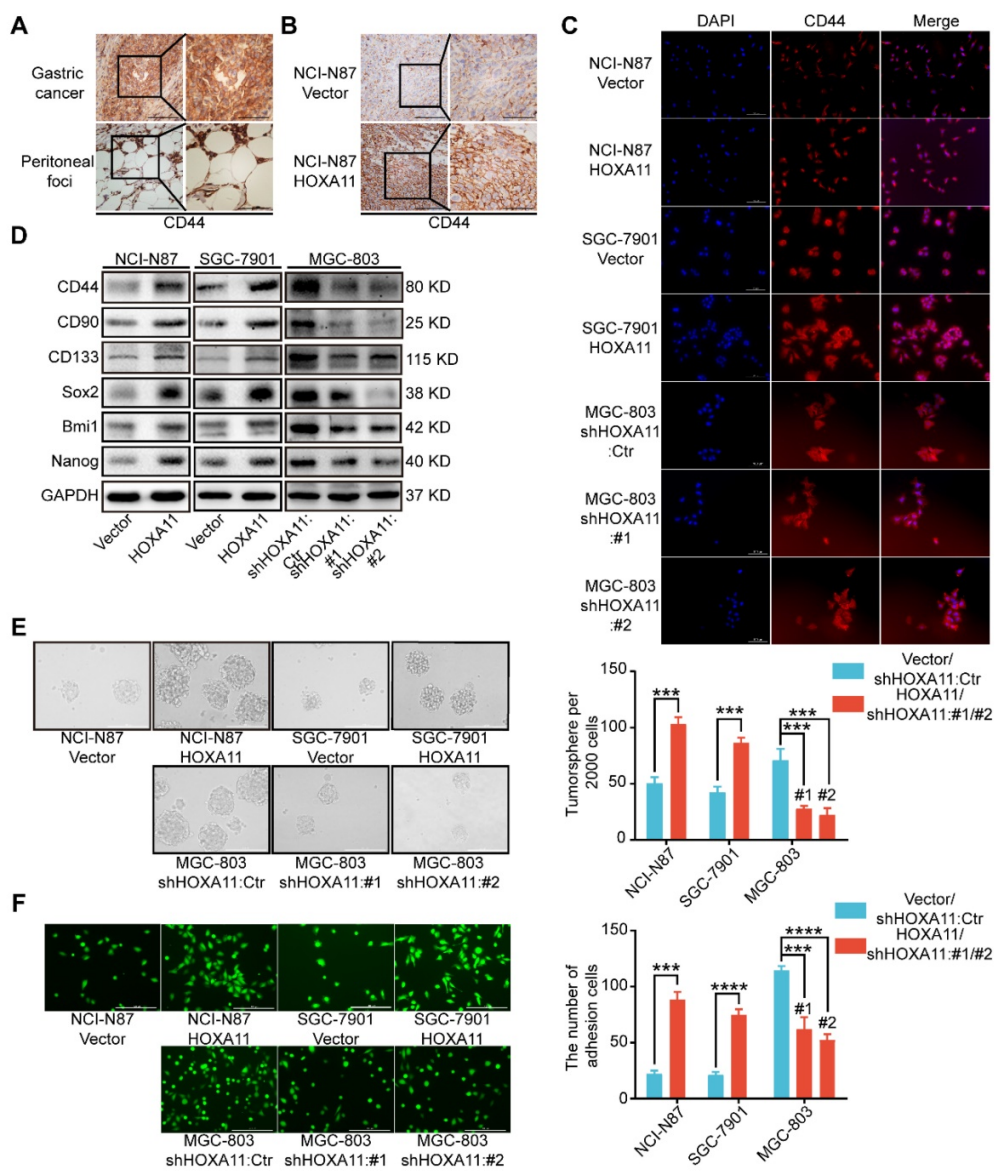
HOXA11 enhanced gastric cancer cell stemness and adhesion. (A) Immunohistochemistry assay shown the expression of CD44 in both primary gastric cancer and peritoneal foci, the left scale bar, 200 μm, 200× magnification; the right scale bar, 100 μm, 400× magnification. (B) Immunohistochemistry assay shown the expression of CD44 in peritoneal foci derived from NCI-N87-Vector and NCI-N87-HOXA11 cell groups, the left scale bar, 200 μm, 200× magnification; the right scale bar, 100 μm, 400× magnification. (C) Immunofluorescence staining for CD44 in NCI-N87-Vector, NCI-N87-HOXA11, SGC-7901-Vector, SGC-7901-HOXA11, MGC-803-Control, MGC-803-shHOXA11 #1 and MGC-803-shHOXA11 #2 cells were shown here (CD44, red; DAPI, blue). The scale bar. 100 μm, 200× magnification; each experiment was performed in triplicate. (D) The protein expression of CD44, CD90, CD133, Sox2, Bmi1 and Nanog in NCI-N87-Vector, NCI-N87-HOXA11, SGC-7901-Vector, SGC-7901-HOXA11, MGC-803-Control, MGC-803-shHOXA11 #1 and MGC-803-shHOXA11 #2 cells were analyzed using western blot with the indicated antibodies. GAPDH was used as the internal protein loading control. Each experiment was performed in triplicate. (E) Representative images of tumorsphere formed by the indicated cells in suspension with cancer stem cell medium. Histograms shown the mean number of tumorsphere. The scale bar, 400 μm, 100× magnification. Results were shown as mean ± SEM of three independent experiments, each experiment was performed in triplicate. ***, P<0.001 (Student* t* test and the analysis of variance test). (F) Representative images of adhesion assays which gastric cancer cells adhere to the HPMC. Histograms shown the number of adhered gastric cancer cells in each group. The scale bar, 400 μm, 100× magnification. Results were shown as mean ± SEM of three independent experiments, each experiment was performed in triplicate. ***, P<0.001; ****, P<0.0001 (Student* t* test and the analysis of variance test).

Stemness have been reported to be closely associated with tumor metastasis, especially in gastric cancer 14-18. Immunohistochemistry assay were performed to examine the cancer stem cell markers in primary gastric cancer and peritoneal foci, we found that CD44 is highly expressed in both sites, and the expression is strongly positive in peritoneal ones especially (Figure 3A). To investigate whether stemness could participate in the process of peritoneal metastasis directed by HOXA11, we stained the tissue sections with CD44 by IHC technology and observed that CD44 is more highly expressed in the “HOXA11 group” than that in “control ones” (P<0.001; Figure 3B and Figure S1J). To perform proof-of-principle *in vitro* study, we have done the immunofluorescence assay to examine the expression of CD44 in gastric cancer cells which HOXA11 was overexpressed or knocked down, the results have shown that CD44 is upregulated in gastric cancer cells which HOXA11 was induced, by contrast, CD44 is downregulated in MGC-803 cells which HOXA11 was knocked down (Figure 3C). We wonder if HOXA11 might be directly involved in stemness maintenance, to investigate this possibility, we analyzed expression of critical cancer stem cell factors and pluripotency markers. Very fascinatingly, we found that upregulation of HOXA11 in gastric cancer cells resulted in increased expression of cancer stem cell factors such as CD44, CD90, CD133 and Bmi1 as well as pluripotency markers Nanog and Sox2 and vice versa (Figure 3D and Figure S1H). In addition to distinct gene expression profiles, stemness can be measured by the ability to form tumorsphere when cancer cell cultured in stem cell media 15. Quantifying the number of tumorsphere in culture, and confirming that overexpression of HOXA11 can enhance tumorsphere-forming capacity in gastric cancer cells, conversely, knockdown of endogenous HOXA11 in MGC-803 cells led to a decrease in tumorsphere numbers (NCI-N87: P<0.001, SGC-7901: P<0.001 and MGC-803: #1: P<0.001 and #2: P<0.001; Figure 3E).

### HOXA11 promoted gastric cancer cells adherence to peritoneal mesothelial cells

It is to be noted that CD44 is not only one type of cancer stem cell markers^19^, but also functionally involved in cell-cell adhesion, even interlink extracellular matrixes with intracellular signaling networks 20, whilst, before the disseminated gastric cancer cells could form the peritoneal metastasis successfully, they must adhere to the peritoneal mesothelium tightly. The adhesion assay was performed to test whether the affinity of gastric cancer cells for peritoneal mesothelium were affected by HOXA11, and significant differences were detected (Figure 3F). Overexpression of HOXA11 in NCI-N87 and SGC-7901 parental cells caused a marked increase in the capacity of cells to adhere to human peritoneal mesothelium cells (HPMC). (NCI-N87: P<0.001; SGC-7901: P<0.0001). Correspondingly, downregulation of HOXA11 in MGC-803 parental cells reduced adhesion to HPMC to the levels shown by parental shControl cells (MGC803: #1: P<0.001 and #2: P<0.0001), confirming that HOXA11 promote gastric cancer cells to adhere to the peritoneal mesothelium.

### HOXA11 suppressed apoptosis and anoikis of gastric cancer cells

**Figure 4:**
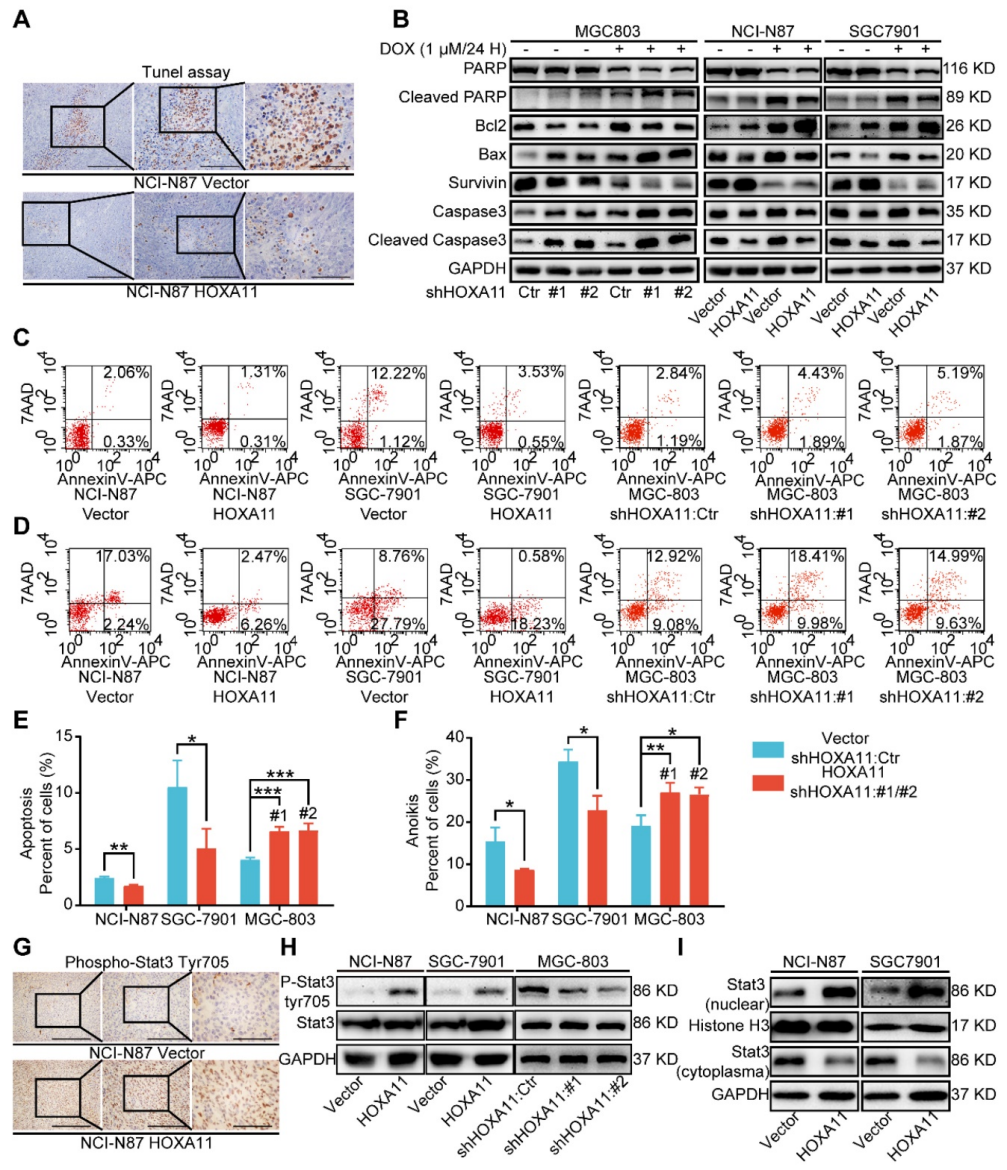
HOXA11 suppressed apoptosis and anoikis of gastric cancer cells and activated Stat3 signaling pathways. (A) Representative images of Tunel assays in peritoneal foci derived from NCI-N87-Vector and NCI-N87-HOXA11 cell groups. The scale bar, from left to right, 400 μm, 100× magnification; 200 μm, 200× magnification; 100 μm, 400× magnification. Each experiment was performed in triplicate. (B) The protein expression of PARP, Cleaved PARP, Bcl2, Bax, Survivin, Caspase3, and Cleaved caspase3 in NCI-N87-Vector, NCI-N87-HOXA11, SGC-7901-Vector, SGC-7901-HOXA11, MGC-803-Control, MGC-803-shHOXA11 #1 and MGC-803-shHOXA11 #2 cells treated with Doxorubicin (1 μm) for 24 hours or not were analyzed using western blot with the indicated antibodies. GAPDH was used as the internal protein loading control. Each experiment was performed in triplicate. (C) The apoptosis of indicated cells were analyzed by flow cytometry via labeling with APC-Annexin-V and 7-AAD. Representative images were shown. (D) The anoikis of indicated cells were analyzed by flow cytometry via labeling with APC-Annexin-V and 7-AAD. Representative images were shown. (E & F) Histograms shown the apoptosis and anoikis level of indicated cells respectively. Results were shown as mean ± SEM of three independent experiments, each experiment was performed in triplicate. *, P<0.05; **, P<0.01; ***, P<0.001 (Student* t* test and the analysis of variance test). (G) Immunohistochemistry assays shown the expression of phosphorylation (Tyr705) of Stat3 in peritoneal foci derived from NCI-N87-Vector and NCI-N87-HOXA11 cell groups. The scale bar, from left to right, 400 μm, 100× magnification; 200 μm, 200× magnification; 100 μm, 400× magnification. Each experiment was performed in triplicate. (H) The protein expression of phosphorylation (Tyr705) of Stat3 and Stat3 in NCI-N87-Vector, NCI-N87-HOXA11, SGC-7901-Vector, SGC-7901-HOXA11, MGC-803-Control, MGC-803-shHOXA11 #1 and MGC-803-shHOXA11 #2 cells were analyzed using western blot with the indicated antibodies. GAPDH was used as the internal protein loading control. Each experiment was performed in triplicate. (I) HOXA11 overexpression increased Stat3 nuclear accumulation in indicated cells, as confirmed by western blot analysis. GAPDH and Histone H3 were used as loading controls. Each experiment was performed in triplicate.

Cell death may occur by anoikis due to the absence of cell attachment, and stemness have been related to resistance to anoikis or apoptosis 21, 22. Tunel assay was used to detect the apoptosis status of peritoneal foci in mice. As shown in Figure 4A and Figure S1K, the apoptosis index of the HOXA11 group was much lower than that in the control ones (P<0.0001), accompanied by upregulated expression of Survivin and Bcl2 as well as downregulated expression of cleaved PARP, cleaved caspase 3 and Bax in NCI-N87 and SGC-7901 cells which HOXA11 was induced, by contrast, downregulation of HOXA11 decreased the expression of Survivin and Bcl2, and even elevated the expression of cleaved PARP, cleaved caspase 3 and Bax in MGC-803 cells (Figure 4B). Apoptosis or anoikis, tested *in vitro* by culturing cells in attachment or suspension respectively, were reduced in HOXA11 high-expressed gastric cancer cells compared to the control ones (Apoptosis: NCI-N87: P<0.01, SGC-7901: P<0.05; anoikis: NCI-N87: P<0.05, SGC-7901: P<0.05), correspondingly, Apoptosis or anoikis were increased in MGC-803 cells which HOXA11 was knocked down (Apoptosis: #1: P<0.001 and #2: P<0.001; anoikis: #1: P<0.01 and #2: P<0.05), above results indicated that HOXA11 could suppress apoptosis and anoikis of gastric cancer cells.

### HOXA11 activated Stat3 signaling pathway

Since Stat3 signaling pathway was one of the most crucial pathways in maintaining the expression of genes that are essential for cancer stemness phenotype, and frequently activated in gastric cancer 17, 23, 24. When located in the cytoplasm, Stat3 protein served as inactive protein, and phosphorylation of tyrosine residue (Tyr) 705 could activate Stat3, which then be translocated to the nucleus. Stat3 worked as a transcription factor and bounded to consensus response elements in the promoters of target genes in the nucleus, Furthermore, it induced the transcription of a broad panel of genes, leading to initiation of signaling cascades for angiogenesis, metastasis, anit-apoptosis, even immune escape in cancerous cells 25. Immunohistochemistry assay was adapted to detect the phosphorylation status of Stat3 in peritoneal foci of mice, and compared with “control group”, the staining intensity of phosphorylation (Tyr705) was obviously stronger in “HOXA11 group” (P<0.0001; Figure 4G and Figure S1L). To perform proof-of-principle *in vitro* study, western blotting was used to detect the phosphorylation (Tyr705) of Stat3 and distribution of Stat3 in cytoplasm and nucleus. The results have shown that HOXA11 can enhance the phosphorylation (Tyr705) of Stat3 and induce Stat3 nuclear accumulation (Figure 4H & I).

### HOXA11 modulated malignant phenotypes which was required for transcriptional activation of Stat3

**Figure 5:**
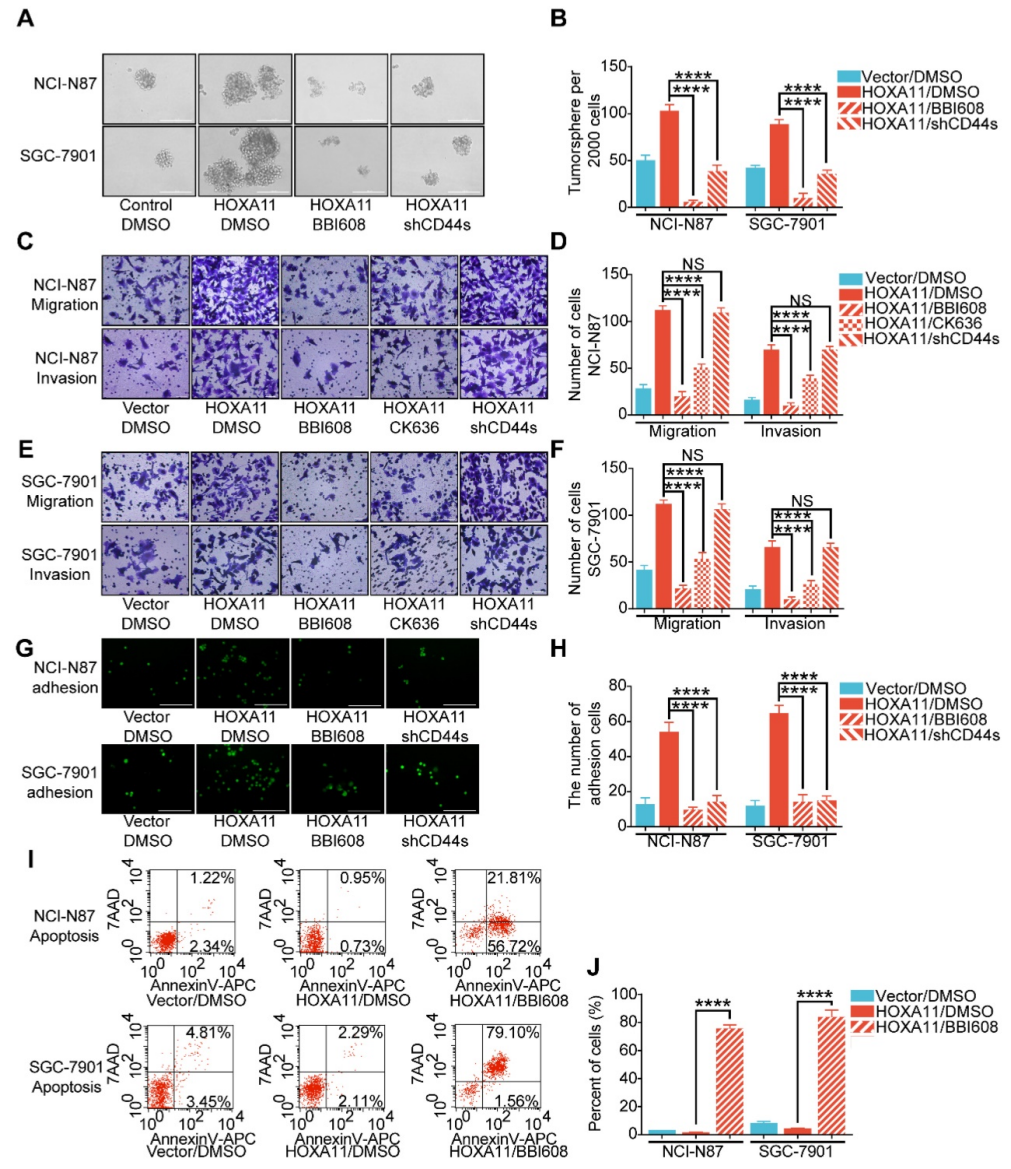
HOXA11 mediated malignant phenotypes which was required for transcriptional activation of Stat3. (A & B) Representative images of tumorsphere formed by the indicated cells in suspension with cancer stem cell medium and then treated with either DMSO or BBI608 (NCI-N87-HOXA11 cells: 3 μM or SGC-7901-HOXA11 cells: 6.5 μM) for 24 h at 37°C. Histograms shown the mean number of tumorsphere. The scale bar, 400 μm, 100× magnification. Results were shown as mean ± SEM of three independent experiments, each experiment was performed in triplicate. ****, P<0.0001 (the analysis of variance test). (C to F) Representative images of cell migration and invasion assays were performed to investigate the indicated cells treated with knockdown of CD44s, BBI608 (NCI-N87-HOXA11 cells: 3 μM or SGC-7901-HOXA11 cells: 6.5 μM), CK636 (4 μM) or DMSO for 24 h at 37°C. The scale bar, 120 μm, 200× magnification. Histograms shown the number of migratory/invading cells. Results were shown as mean ± SEM of three independent experiments, each experiment was performed in triplicate. NS, no significance; ***, P<0.001; ****, P<0.0001 (the analysis of variance test). (G & H) Representative images of adhesion assays which the indicated cells treated with knockdown of CD44s, BBI608 (NCI-N87-HOXA11 cells: 3 μM or SGC-7901-HOXA11 cells: 6.5 μM) or DMSO for 24 h at 37°C, and then trypsinized into single suspension and adhered to the HPMC for 3 h. Histograms shown the number of adhered gastric cancer cells in each group. The scale bar, 400 μm, 100× magnification. Results were shown as mean ± SEM of three independent experiments, each experiment was performed in triplicate. ****, P<0.0001 (the analysis of variance test). (I & J) The apoptosis of indicated cells treated with BBI608 (NCI-N87-HOXA11 cells: 3 μM or SGC-7901-HOXA11 cells: 6.5 μM) or DMSO for 24 h at 37°C were analyzed by flow cytometry via labeling with APC-Annexin-V and 7-AAD. Representative images were shown. Histograms shown the apoptosis level of each group respectively. Results were shown as mean ± SEM of three independent experiments, each experiment was performed in triplicate. ****, P<0.0001 (the analysis of variance test).

The next step was to investigate whether HOXA11 regulate malignant phenotypes is required for the induction of Stat3 activity and elucidate the signaling mechanism. The results of tumorsphere formation assay have shown that the number of tumorsphere is decreased when Stat3 is suppressed by Napabucasin (BBI608), which could inhibit gene transcription driven by Stat3 and cancer stemness properties 15 (NCI-N87: P<0.0001; SGC-7901: P<0.0001), besides, knockdown of mesenchymal splice isoform of CD44 (CD44s) could also inhibited the formation of tumorsphere (NCI-N87: P<0.0001; SGC-7901: P<0.0001; Figure 5A & B). As shown in Figure 5C-F, migration and invasion ability of gastric cancer cell which HOXA11 was induced could be abolished by suppressing Stat3 activity (NCI-N87: migration: P<0.0001, invasion: P<0.0001; SGC-7901: migration: P<0.0001, invasion: P<0.0001), knockdown of CD44s failed to reduce the number of cells passed through the membrane which were increased by HOXA11, moreover, application of CK636, an small molecule inhibitor of the Arp2/3 complex which regulate the invadopodia-based invasion 26, diminished the migration and invasion of gastric cancer cells enhanced by HOXA11 (NCI-N87: migration: P<0.0001, invasion: P<0.0001; SGC-7901: migration: P<0.0001, invasion: P<0.0001), confirmed that HOXA11 modulate the migration and invasion of gastric cancer cells via Stat3 and Arp2/3 complex-dependent mechanism. Akin to stemness, the adhesive ability of gastric cancer cells induced by HOXA11 were blocked by BBI608 or knockdown of CD44s respectively (NCI-N87: BBI608: p<0.0001, shCD44s: P<0.0001; SGC-7901: BBI608: P<0.0001, shCD44s: P<0.0001; Figure 5G & H). Furthermore, compared with the equivalent control concentration of DMSO, BBI608 rescued the apoptosis level of gastric cancer cells which was downregulated by HOXA11 (NCI-N87: P<0.0001; SGC-7901: P<0.0001; Figure 5I & J).

**Figure 6:**
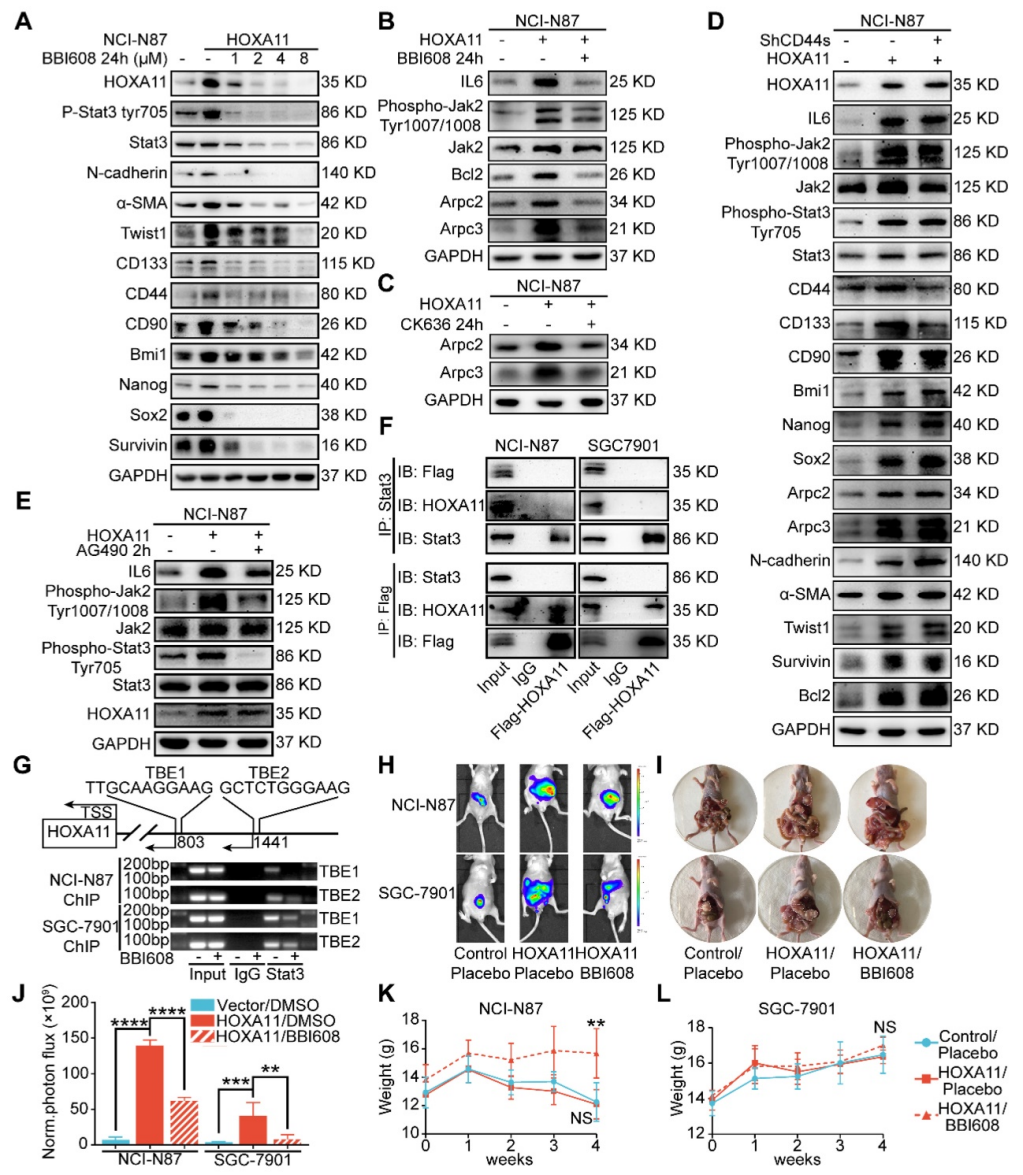
HOXA11-Stat3 generated a positive-feedback loop, and inhibition of Stat3 suppressed the peritoneal metastasis caused by HOXA11. (A) NCI-N87-HOXA11 cells were treated with BBI608 at a density of 1, 2, 4, or 8 μM or with DMSO (-) as well as NCI-N87-Vector cells treated with DMSO (-) for 24 h at 37°C. Cell lysates from the indicated cells were then analyzed by western blot. BBI608 suppressed the expression of HOXA11, crucial metastatic and stemness related proteins. GAPDH was used as the internal protein loading control. Each experiment was performed in triplicate. (B) The protein expression of IL6, phosphorylation (Tyr1007/1008) of Jak2, Jak2, Bcl2, Arpc2 and Arpc3 in NCI-N87-Vector and NCI-N87-HOXA11 cells treated with BBI608 (3 μM) for 24 h at 37°C were analyzed using western blot with the indicated antibodies. GAPDH was used as the internal protein loading control. Each experiment was performed in triplicate. (C) The protein expression of Arpc2 and Arpc3 in NCI-N87-Vector and NCI-N87-HOXA11 cells treated with CK636 (4 μM) for 24 h at 37°C were analyzed using western blot with the indicated antibodies. GAPDH was used as the internal protein loading control. Each experiment was performed in triplicate. (D) NCI-N87-HOXA11 cells were treated with knockdown of CD44s for 24 h at 37°C. Cell lysates from the indicated cells were then analyzed by western blot with the indicated antibodies. GAPDH was used as the internal protein loading control. Each experiment was performed in triplicate. (E) NCI-N87-HOXA11 cells were treated with AG490 (60 μM) for 2 h at 37°C. Cell lysates from the indicated cells were then analyzed by western blot with the indicated antibodies. GAPDH was used as the internal protein loading control. Each experiment was performed in triplicate. (F) Co-IP experiments in NCI-N87-flag-HOXA11 and SGC-7901-flag-HOXA11 cells using anti-Stat3 and anti-Flag antibodies, respectively. (G) The ChIP-qPCR experiments were performed to assess whether the binding ability of Stat3 on the promoter region of HOXA11 which was affected by BBI608, a schematic illustrated the relative positions of qPCR probes to putative transcription binding elements (TBEs) for ChIP-qPCR experiments. TBE1: TTGCAAGGAAG, TBE2: GCTCTGGGAAG. IgG was used as a negative control. (H & I) BBI608, which targets Stat3, suppressed the peritoneal metastasis of gastric cancer cells caused by HOXA11 in BALB/c mice. Tumor in all groups were measured both in situ and after laparotomy. (I) Statistical analysis of the bioluminescence in peritoneal foci of all groups. Results were shown as mean ± SEM, **, P<0.01; ***, P<0.001; ****, P<0.0001 (the analysis of variance test). (K & L) These mice were given BBI608 (20 mg/kg), or DMSO i.p. All regimens were administered twice a week. Body weight was measured weekly during the treatment. There was no significant decrease in body weight due to administration of the BBI608.

To elucidate the mechanism by which HOXA11 modulates the malignant phenotype more intuitively, immunoblots from gastric cancer cell lysates treated with BBI608 have shown that BBI608 treatment resulted in a dose-dependent decrease in the HOXA11, phosphorylation level of Stat3, Stat3 itself and protein markers regarding cancer stemness (CD133, CD44, CD90, Bmi1, Nanog and Sox2), metastasis (N-cadherin, α-SMA and Twist1) and survival (Survivin) (Figure 6A and Figure S2A). To expand on this observation, the expression of Arpc2 and Arpc3, the subunits of Arp2/3, in the gastric cancer cells which had been treated with BBI608 or not were examined and it was found that BBI608 could downregulate the protein level of Arpc2 and Arpc3 upregulated by HOXA11. HOXA11 enhanced the expression of Arpc2 and Arpc3 via mediating the activation of Stat3 (Figure 6B and Figure S2B). Moreover, CK636, the small molecule inhibitor of Arp2/3 complex which has been confirmed to participate in the process by which HOXA11 regulates the migration and invasion of gastric cancer cells, could also downregulate the expression of Arpc2 and Arpc3 which were upregulated by HOXA11 (Figure 6C and Figure S2C). Besides, knockdown of CD44s has no effect on the expression of metastatic related markers and only downregulated the expression of CD44 and CD133 (Figure 6D and Figure S2D).

### HOXA11-Stat3 generated a positive-feedback loop

As shown in Figure 6B and Figure S2B, HOXA11 upregulated the expression of IL6 and phosphorylation of Tyr 1007/1008 on Jak2, which are the upstream activator of Stat3. This tendency could be diminished by BBI608 when treated with NCI-N87-HOXA11 and SGC-7901-HOXA11 cells at IC50 dosage. Furthermore, the IC50 of the inhibitory activity of BBI608 in gastric cancer cells which HOXA11 was induced (NCI-N87-HOXA11: 3.0 μM; SGC-7901-HOXA11: 6.5 μM) were higher than their control ones (NCI-N87-Vector: 0.4 μM; SGC-7901-Vector: 2.8 μM) (Figure S2F). Stat3 have been reported to bind to the IL6 promoter and generate a positive-feedback loop which lead to increase expression of IL6 25. To test whether HOXA11 take part in the positive-feedback loop of IL6-Jak2-Stat3, AG490, selectively inhibited JAK/Stat activation 27, have been used to treat with NCI-N87-HOXA11 and SGC-7901-HOXA11 cells at dosage of 60 μM, and it was found that the expression of HOXA11 is not altered by AG490 (Figure 6E and Figure S2E). HOXA11 did not participate in the loop of IL6-Jak2-Stat3, albeit HOXA11 and Stat3, both of which belong to transcription factors and might serve as co-transcription factors in nucleus, Reciprocal co-immunoprecipitation experiment with anti-Stat3 and anti-Flag antibodies in NCI-N78-flag-HOXA11 and SGC-7901-flag-HOXA11 cell lines were performed to test whether HOXA11 interact with Stat3 and the results have shown that HOXA11 could not interact with Stat3 (Figure 6F). To investigate if Stat3 may directly interact with the HOXA11 gene promoter element, the full length HOXA11 promoter (27185217-27187216; 2 Kb) was cloned into a luciferase reporter plasmid (Figure S2G), and was then co-transfected with empty or Stat3-containing plasmids into NCI-N87 and SGC-7901 cells and the resultant relative light units (RLU) have shown a robust and positive response induced by Stat3. Furthermore, the binding sites of transcription factor Stat3 in the promoter zone of HOXA11 have been predicted via the JASPAR database 28 and to confirm that Stat3 activates HOXA11 expression via transcription binding element (TBE) 1 and 2, we generated luciferase reporters containing HOXA11 promoter with mutation of binding element (TBE) 1, 2 or 1&2 as indicated, and induction of HOXA11 luciferase reporters were completely abolished (Figure S2H). To substantiate a putative interaction between the TBE 1&2 and Stat3, the genomic occupancies of Stat3 at the TBE 1&2 were verified by chromatin immunoprecipitation followed by qPCR (ChIP-qPCR) (Figure 6G), moreover, the binding ability of Stat3 on the promoter of HOXA11 could be inhibited by BBI608 treated with NCI-N87-HOXA11 and SGC-7901-HOXA11 cells at the dosage of 3.0 μM and 6.5 μM respectively (Figure 6G & Figure S2I). Collectively, these experiments indicated that HOXA11 is the target gene of transcription factor Stat3, and both of them form a positive-feedback loop regulating the malignant phenotype of gastric cancer cells.

### HOXA11 promoted peritoneal metastasis via activating Stat3 in xenograft mice models

To determine the peritoneal metastatic promotion of HOXA11* in vivo*, the xenograft mice models were established by injecting NCI-N87-HOXA11, NCI-N87-Vector, SGC-7901-Vector and SGC-7901-HOXA11, MGC-803-Contrl, MGC-803-shHOXA11 #1 and MGC-803 shHOXA11 #2 cells into the abdomen of BALB/c mice, and four days after injection, BBI608 should be, subsequently injected into the abdomen of mice in which group had been injected with NCI-N87-HOXA11, SGC-7901-HOXA11 and MGC-803-Contrl cells, and other ones treated with placebo. The tumor burdens of HOXA11 high-expressed groups were heavier than that of the controls and BBI608 treated groups in the peritoneum of hosts (NCI-N87: Vector/DMSO vs. HOXA11/DMSO: P<0.0001 and HOXA11/DMSO vs. HOXA11/BBI608: P<0.0001; SGC-7901: Vector/DMSO vs. HOXA11/DMSO: P<0.001 and HOXA11/DMSO vs. HOXA11/BBI608: P<0.01 and MGC-803: Vector/DMSO vs. Vector/BBI608: P<0.0001, Vector/DMSO vs. shHOXA11 #1/DMSO: P<0.0001 and Vector/DMSO vs. shHOXA11 #2/DMSO: P<0.0001; Figure 6H-J and Figure S3A-B), moreover, no signs of toxicity as evidenced by body weight measurement were observed (Figure 6K & L and Figure S3C). The peritoneal foci were extracted and then processed into paraffin embedded tissue sections. Hematoxylin-eosin (H&E) staining separated component of tumor from mesothelioma clearly, we stained the serial tissue sections with HBME-1 and Calretitin which are the markers of mesothelioma (Figure S3D), and found that the expression of HBME-1 and Calretitin in BBI608 treated groups were stronger than that in placebo treated ones (HBME-1: NCI-N87: P<0.001 and SGC-7901: P<0.001; Calretitin: NCI-N87: P<0.0001 and SGC-7901: P<0.0001; Figure S3E-F). It has to be noted that tumor components are engulfed by mesothelioma in BBI608 treated groups, which indicate that the remaining peritoneal foci might be protected by mesothelioma to defend against the killing effect of BBI608.

### HOXA11 was frequently upregulated in gastric cancer tissues and its correlation with clinicopathological parameters

**Figure 7:**
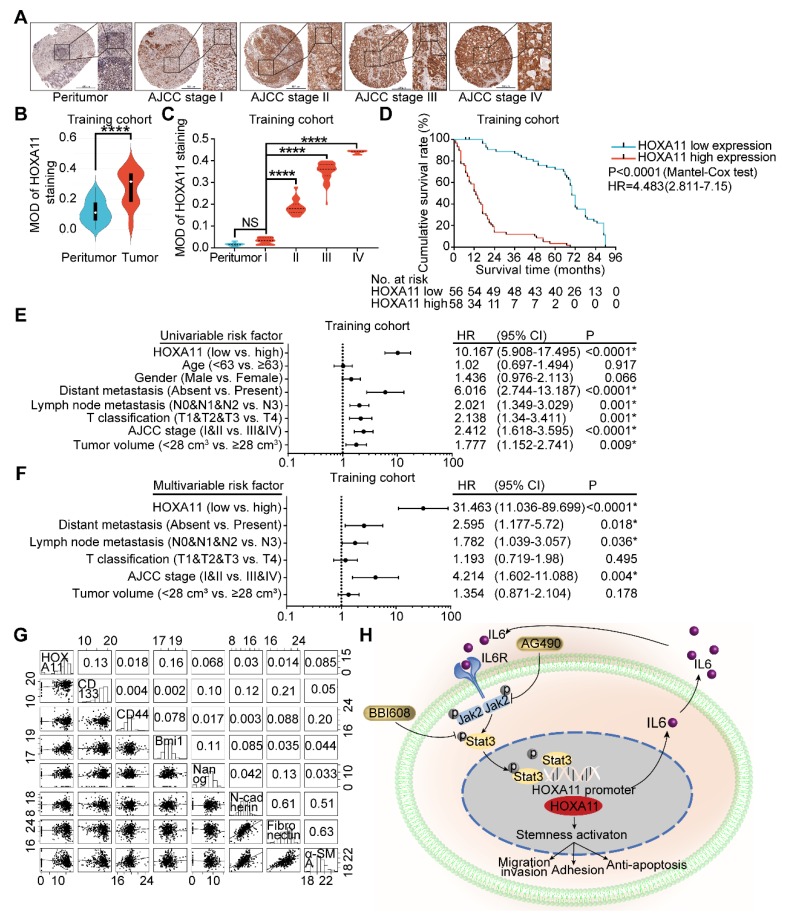
HOXA11 was frequently upregulated in gastric cancer tissues and its correlation with clinicopathological parameters. (A) Examination of HOXA11 expression in primary gastric cancer and peritumor tissues by IHC. The scale bar, 500 μm, 50× magnification; 100 μm, 200× magnification; 50 μm, 400× magnification; (B) Statistical analysis of the HOXA11 expression in the paired primary gastric cancer and peritumor tissues of training cohort by MOD of staining. ****, P<0.0001 (Student *t* test). (C) Statistical analysis of the HOXA11 expression in peritumor tissues of AJCC stage I and primary gastric cancer tissues of different AJCC stages of training cohort by MOD of staining. NS, no significance; ****, P<0.0001 (the analysis of variance test). (D) Survival of patients in HOXA11-low expression group and HOXA11-high expression group. The survival time of patients in the training cohort was compared between groups using the Mantel-Cox test, which presented significantly longer survival of patients in the HOXA11-low expression group (P<0.0001). (E) Univariate analysis was performed in training cohort. The bar corresponds to 95% confidence intervals. (F) Multivariate analysis was performed in training cohort. The bar corresponds to 95% confidence intervals. (G) Correlations among HOXA11, CD133, CD44, Bmi1, Nanog, N-cadherin, Fibronectin and α-SMA levels in human gastric cancer tissues (TCGA, n=375). (H) Schematic diagram of the relationship between HOXA11, stemness, migration and invasion, adhesion and anti-apoptosis.

**Figure 8:**
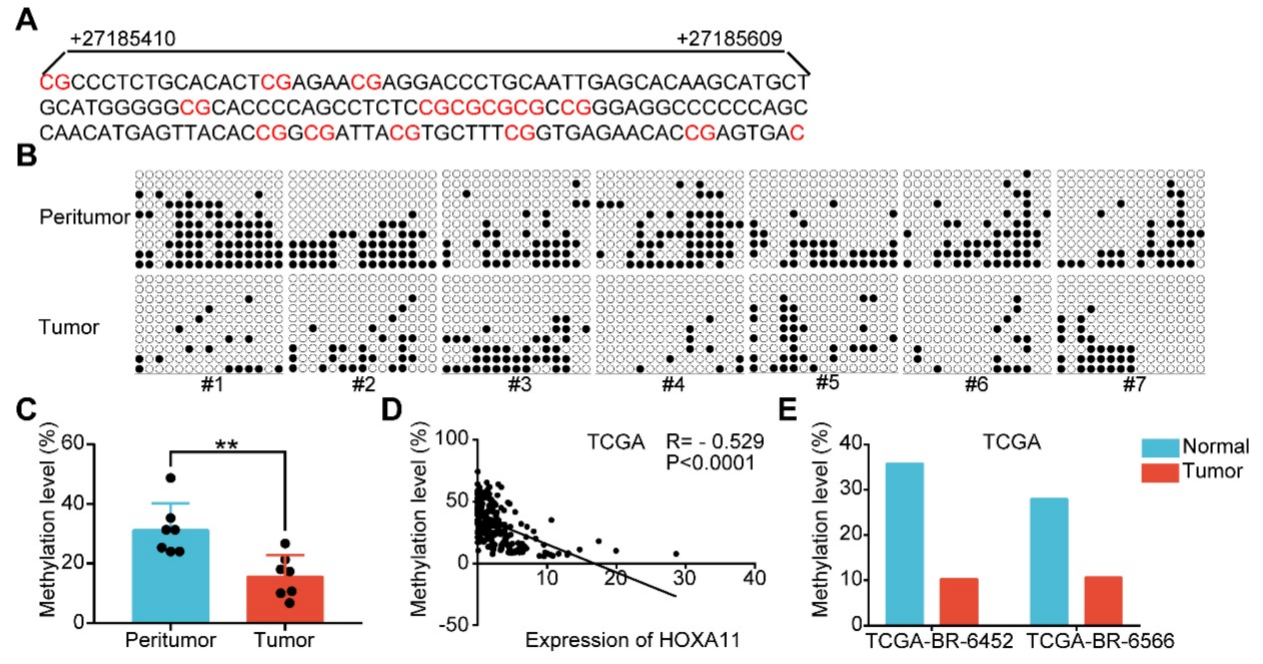
HOXA11 expression and promoter methylation in primary gastric cancer tissues and peritumor normal tissues. (A) The schematic illustration of 15 CpG sites located between nucleotides +27185410-+27185609 in the HOXA11 promoter for BSP. (B & C) BSP was performed to evaluate HOXA11 CpG island methylation statuses in seven paired gastric cancer tissues and peritumor normal tissues. All images and corresponding statistical plots were presented. Filled circles and open circles represent methylated and unmethylated CpG sites, respectively. (D) The correlation between expression of HOXA11 and methylation level of HOXA11 promoter in TCGA database. (E) The methylation level of HOXA11 promoter in two paired gastric cancer tissues and peritumor normal tissues (TCGA-BR-6452 & TCGA-BR-6566).

To ascertain the manner of expression of HOXA11 in gastric cancer, tumor tissue and matched peritumor specimens of gastric cancer patients with different clinico-pathological features were examined by IHC staining, and the overall cohort of 190 gastric cancer patients were subdivided into a training set (n=114) and a validation set (n=76). The HOXA11 was located in the nucleus with variable staining intensity, and the staining intensity was stronger as the AJCC stage was higher (Figure 7A & C and Figure S4B). Furthermore, compared with the tumor tissues, HOXA11 expression was lower in peritumor tissues (training cohort: P<0.0001; validation cohort: P<0.0001; Figure 7B and Figure S4A), this tendency had also been validated by the mRNA expression level of HOXA11 in GSE13861, GSE13911 and TCGA database. We observed that HOXA11 expression are significantly higher in tumor than that in normal tissues (GSE13861: P<0.0001, GSE13911: P<0.0001 and TCGA: P<0.0001; Figure S4C-E). The gastric cancer patients in training cohort and validation ones were then stratified according to MOD of HOXA11 staining (the cutoff value was 0.31), Kaplan-Meier survival curves showed that gastric cancer patients with higher HOXA11 expression have significantly shorter overall survival (OS) in training cohort (P<0.0001, Figure 7D), validation ones (P<0.0001, Figure S4F) and GEO database from the Kaplan-Meier plotter (www.Kmplot.com) (P<0.0001, Figure S4G). Statistical analysis of the correlation between HOXA11 expression and clinico-pathological characteristics and revealed that higher HOXA11 expression is associated with advanced AJCC stage (P<0.0001 & P<0.0001), advanced T phase of tumor (P<0.0001 & P<0.0001), advanced lymph node metastasis (P<0.0001 & P<0.0001) and larger tumor volume (P=0.024 & P=0.006) in training cohort and validation ones respectively (Table S2-3). In addition, higher HOXA11 expression was associated with distant metastasis in the training cohort (P=0.006) (Table S2). It is, thus, of interest to investigate whether HOXA11 expression level is an independent prognostic risk factor for gastric cancer patients, COX regression hazard model was used to perform univariate and multivariate analysis, and the results, which adjust lymph node metastasis, AJCC stage and other factors confirmed the association between HOXA11 expression and shorter survival (hazard ration (HR): 10.167; 95% confidence interval (CI): 5.908-17.495; P<0.0001 & HR: 31.463; 95% CI: 11.036-89.699; P<0.0001; respectively; Figure 7E & F), and further validated in validation cohort (HR: 9.006; 95% CI: 4.732-17.141; P<0.0001 & HR: 25.372; 95% CI: 7.309-88.073; P<0.0001, respectively; Figure S4H & I). To generate a more accurate predictive model, the independent prognostic risk factors in training cohort were used to establish a prognostic nomogram. The C-index for OS prediction of the formulated nomogram in training cohort was 0.767 (95% CI: 0.734-0.800; P<0.0001), as shown in Figure S5A, the nomogram predicting 3- and 5- year overall survival that were constructed based on selected risk factors with hazard ratios. The nomogram could calculate the probability of survival via adding up the scores identified on the points scale for each risk factor. The total score projected to the bottom scale demonstrated the probability of 3- and 5- year survival. The calibration plot for the probability of 3- and 5- year survival revealed that optimal agreement between the prediction by nomogram and actual observation (Figure S5B-C). The predictive accuracy of the nomogram for OS was further validated in the validation cohort. The C-index of the constructed nomogram derived from training cohort for OS prediction in the validation ones was 0.765 (95% CI: 0.719-0.811; P<0.0001), moreover the calibration curve fitted well between the prediction by nomogram and actual observation in the probability of 3- and 5- year survival (Figure S5D-E). The mRNA expression of HOXA11 was positively correlated with the expression level of CD133 (R=0.13, P=0.014) and Bmi1 (R=0.16, P=0.0023) in gastric cancer tissues of TCGA database (Figure 8G). Collectively, the data demonstrated that HOXA11 and Stat3 form a positive-feedback loop which enhances the stemness of gastric cancer cells, subsequently resulting in promotion of migration and invasion, adhesion and resistance to apoptosis (Figure 7H).

### Promoter methylation was involved in HOXA11 downregulation in gastric cancer

Highly dense CpG islands were common in most of the HOXA11 promoters and the hypermethylation of these CpG islands could regulate HOXA11 gene expression in non-small cell lung cancer 29, glioblastoma 30 and renal cell carcinoma 5. The methylation status of 15 CpG sites at the promoter region of HOXA11 were analyzed using bisulfite sequencing PCR (BSP) in seven paired gastric cancer tissues along with their matched peritumor normal tissues, and it was found that peritumor normal tissues have markedly greater methylation level than their matched ones (P<0.01; Figure 8A-C). We evaluated the methylation level and expression of HOXA11 in TCGA gastric cancer database using MethHC (http://methhc.mbc.nctu.edu.tw/php/index.php) 31, the results revealed that hypermethylation of promoter decrease the HOXA11 expression (R=-0.529, P<0.0001; Figure 8D), furthermore, methylation data of the only two paired gastric cancer tissues and matched peritumor normal tissues was consistent with BSP result very well (Figure 8E). These results indicated that peritumor normal tissues have higher methylation level on the promoter region of HOXA11 than their matched ones and hypermethylation of promoter decrease the HOXA11 expression.

## Discussion

Here we described a new oncogene and a mechanism of regulation of the peritoneal metastasis in gastric cancer. We found, using *in vivo*,* in vitro*, *in silico* and gastric cancer tissue studies, that HOXA11 is a potent activator of peritoneal metastasis in gastric cancer. HOXA11 had the ability to change the stemness property of gastric cancer cells via forming a novel positive feedback loop with Stat3, and through such reprogramming, to modulate the adhesion, migration/invasion and anti-apoptosis phenotype of gastric cancer cells. These were achieved through the HOXA11-Stat3-CD44s, ARP2/3 and Bcl2 signaling axis described here. (Figure 7H).

In gastric cancer, EMT was associated with enhanced capability of cancer cell invasion and metastasis, and the correlation between EMT and the acquisition of stemness properties were described in multiple tumor types 24, 32, 33, Cancer stemness exhibited characteristics similar to those of tissue stem cells, such as self-renewal, differentiation and resistance to apoptosis or anoikis, and were considered to be crucial for the initiation and maintenance of tumors as well as their metastasis 22, 34. Indeed, the function of stemness in cancer was at least superficially similar to the processes that occur during embryonic development with the mesoderm developing into multiple tissue types, whilst pleiotropically acting transcription factors participating in embryogenesis have been reported to confer malignant traits, such as invasiveness and resistance to apoptosis, on cancer cell 35. HOXA11, which belong to transcription factors, was regulated during embryonic development and play a role in female fertility 36. The current state of the art and latest findings shown that expression of HOXA11 is epigenetically repressed by DNA methylation in human gastric cancer 37, and our prior studies have confirmed that knockdown of MTA2 (metastasis associated 1 family, member 2) could decrease the expression of HOXA11 in gastric cancer cells (Figure S1A) 38, there are still gaps in our understanding of the complex processes by which HOXA11 regulates peritoneal metastasis. Persistent activation of Stat3 regulated genes which drives stemness have been demonstrated to be crucial for tumor progression and therefore negatively affecting the prognosis of patients^19^. Thus, targeting Stat3 and consequently preventing cancer stemness might be a promising strategy for cancer therapeutics 15, 23, 39. Napabucasin (BBI608), which is a small molecule inhibitor of Stat3, have been shown to be able to block cancer relapse and metastasis via inhibiting gene transcription driven by Stat3 and cancer stemness properties 15, 40. In the microenvironment of liver metastasis, BBI608 induced myeloid derived suppressor cells (MDSCs) apoptosis, followed by enhancing of the efficacy of chimeric antigen receptor T cells (CAR-T) 41. Besides, BBI608 could reverse the cisplatin-resistance in Non-small cell lung cancer (NSCLC) via targeting of stemness gene expression 42. In clinical practice, BBI608 have hitherto shown variable efficacy in a variety of cancer types, especially in gastric cancer, either as a monotherapy or in combination with conventional chemotherapeutic agents 43. BBI608 combined with weekly paclitaxel in patients with advanced gastric cancer was studied in phase 1 and phase 1b/2 extension study, results from these studies indicated that BBI608 plus weekly paclitaxel could bring benefit to patients 17. Albeit the BRIGHTER trial, which is a randomized, double-blind, phase 3 trial of BBI608 plus weekly paclitaxel versus placebo plus weekly paclitaxel in patients with advanced, previously treated gastric cancer, did not achieve the primary endpoint that combined treatment extend overall survival in the general study population. The promising value and efficacy of BBI608, which was approved by the FDA for gastric or gastroesophageal junction cancer 44, cannot be denied, as the biomarker-selected patient population who can benefit from BBI608 might be not the predefined biomarker (β-catenin) positive subpopulation 45. Our data indicated that HOXA11 regulated adhesion, migration and invasion and anti-apoptosis of gastric cancer cells through modulating Stat3 activation can be abolished by BBI608. To elucidate the more direct mechanism, we show that Stat3 bind on the promoter of HOXA11 and induce the transcription of HOXA11, both of them form a positive feedback loop. BBI608 blocked the binding ability of Stat3 on the promoter of HOXA11, and further diminished the feedback loop between HOXA11 and Stat3, besides, inhibition of Jak2, the upstream signaling regulator of Stat3, by AG490, were observed to be useless in altering the expression of HOXA11. Collectively, the feedback loop formed by HOXA11 and Stat3 was highly sensitive to direct Stat3 inhibition and not sensitive to inhibition of upstream kinases.

As the putative stemness biomarker, CD44 also functioned as a multifunctional cell surface adhesion receptor, and contributed to cell-cell and cell-matrix adhesion, cell migration, epithelial to mesenchymal transition and cancer metastasis 46. CD44 was encoded by 20 exons which experienced alterative splicing dividing into the standard (CD44s) and variant (CD44v) isoforms. CD44s consisted of the first five exons (exons 1-5) and last five exons (exons 16-20) which is expressed by the mesenchymal cells, while CD44v were named as V1-V10 according to the inclusion of exons between exon 5 and 16 47. Variable splicing isoforms have been reported to have specific physiological functions in various kinds of cancer cells, such as CD44s promoted EMT/invasion of ovarian cancer cell, CD44v6 regulated colon cancer metastasis and splicing switch of CD44 from the epithelial isoform (CD44v) to the mesenchymal isoform (CD44s), promoting EMT and consequently inducing lung metastasis of triple negative breast cancer (TNBC) 47-49. Our data shown that knockdown of CD44s block the ability of tumorsphere formation and adhesion in gastric cancer cells which was enhanced by HOXA11 rather than the migration and invasion regulated by HOXA11, and BBI608 blocked the phosphorylation (Tyr705) of Stat3 which were activated by HOXA11 and followed by reduced CD44 expression level. Taken together, the adhesion and stemness ability of gastric cancer cells might be achieved through the HOXA11-Stat3-CD44s signaling axis described here. Whilst, HOXA11-Stat3 regulated migration and invasion in gastric cancer cell relied on the Arp2/3 complex dependent signaling axis, further investigation demonstrated activation of apoptosis signaling pathways in NCI-N87-HOXA11 and SGC-7901-HOXA11 cells treated with Stat3 inhibition as evidenced by downregulation of the anti-apoptosis protein Bcl2.

The microenvironment encompassed all components of a tumor other than the cancer cells themselves 19, 50. The results of *in vivo* study shown that BBI608 could obviously shrink the peritoneal foci formed by gastric cancer cells which HOXA11 was induced, there still remained foci in the abdomen of mice. Immunohistochemistry of serial tissues sections in remaining foci was done and we observed that mesothelium components are enriched in the group treated with BBI608, and tumor components are engulfed by mesothelium resistant to the killing of BBI608. The tumor microenvironment cannot be ignored as numerous studies provided examples of the bidirectional interactions that occur between cancer cells and the nearby stromal cell: cancer cell stimulate the formation of an inflamed stroma, and latter ones reciprocate by enhancing the malignant characteristics of the cancer cells, collectively, forming a potentially self-amplifying positive feedback loop 51. The therapeutic strategy targeting the traits of “seed” (that is, cancer stemness) can diminish 99% of tumor volume, however for the remaining 1% of tumor volume perhaps there needs to be an altering to the “soil” of the foreign tissue microenvironments present at metastatic sites.

## Conclusion

This is the first study to investigate the relationship between HOXA11 and cancer stemness in peritoneal metastasis of gastric cancer, and we have identified a novel positive feed-back loop of HOXA11/Stat3-dependent stemness of gastric cancer cells supporting peritoneal metastasis that can be targeted by BBI608 (Napabucasin), offering hope for new cancer therapies.

## Supplementary Material

Supplementary figures and tables.

## Abbreviations

*HOXA11*: Homoebox A11
*HPMCs*: Human peritoneal mesothelial cells
STR: short tandem repeat
FBS: fetal bovine serum
FFPE: paraffin-embedded
OS: Overall survival
HE: hematoxylin and eosin
Co-IP: Co-immunoprecipitation
PVDF: polyvinylidene fluoride
ChIP: Chromation immunoprecipitation
ANOVA: one-way analysis of variance
SEM: standard error of the mean
GO: Gene ontology
DAVID: annotation, visualization and integrated discovery
shRNA: short hairpin RNA
Tyr: tyrosine residue
MDSCs: myeloid derived suppressor cells
CAR-T: chimeric antigen receptor T
NSCLC: non-small cell lung cancer
TNBC: triple negative breast cancer
