# Supplementary material for: A self-enforcing HOXA11/Stat3 feedback loop promotes stemness properties and peritoneal metastasis in gastric cancer cells
Source: Theranostics. 2019 Oct 14;9(25):7628–47. doi: 10.7150/thno.36277 (PMC6831465; doi:10.7150/thno.36277)
Supplement: Supplementary file 1 — Supplementary figures and tables. [file thnov09p7628s1.pdf]

Supplementary figure 1. (A) Microarray data (E-MTAB-3469) in ArrayExpress database shown metastasis associated 1 family, member 2 (MTA2), which was knocked down in SGC-7901, could regulate the expression of HOXA11. (B & C) Expression of HOXA11 of indicated cells were analyzed using qRT-PCR. Results were shown as mean ± SEM of three independent experiments, each experiment was performed in triplicate. ***, P<0.001; ****, P<0.0001 (Student *t* test and the analysis of variance test). (D) Immunofluorescence staining for HOXA11 in SGC-7901-Vector, SGC-7901-HOXA11, MGC-803-Control, MGC-803-shHOXA11 #1 and MGC-803-shHOXA11 #2 cells were shown here (HOXA11, red; DAPI, blue). The scale bar. 100 μm, 200× magnification; 50 μm, 400× magnification. Each experiment was performed in triplicate. (E) HOXA11 knockdown in HEK 293T cell line was measured by western blot. GAPDH was used as a loading control. Each experiment was performed in triplicate. (F) Cell lysates from the indicated cells were analyzed by western blot with the indicated antibodies. GAPDH was used as the internal protein loading control. Each experiment was performed in triplicate. (#1: 268; #2: 433). (G & H) Cell lysates from the indicated cells were analyzed by western blot with the indicated antibodies. GAPDH was used as the internal protein loading control. Each experiment was performed in triplicate. (I) Immunofluorescence staining for NCI-N87-Vector, NCI-N87-HOXA11, SGC-7901-Vector, SGC-7901-HOXA11, MGC-803-Control, MGC-803-shHOXA11 #1 and MGC-803-shHOXA11 #2 cells were shown here (N-cadherin, red; DAPI, blue). The scale bar. 100 μm, 200× magnification; 50 μm, 400× magnification. Each experiment was performed in triplicate. (J to L) Statistical analysis of CD44, Tunel and phosphorylation (Tyr705) of Stat3 staining intensity (H-score) in both groups. Results were shown as mean ± SEM of three

independent experiments, each experiment was performed in triplicate. ***, P<0.001, ****, P<0.0001 (Student *t* test).

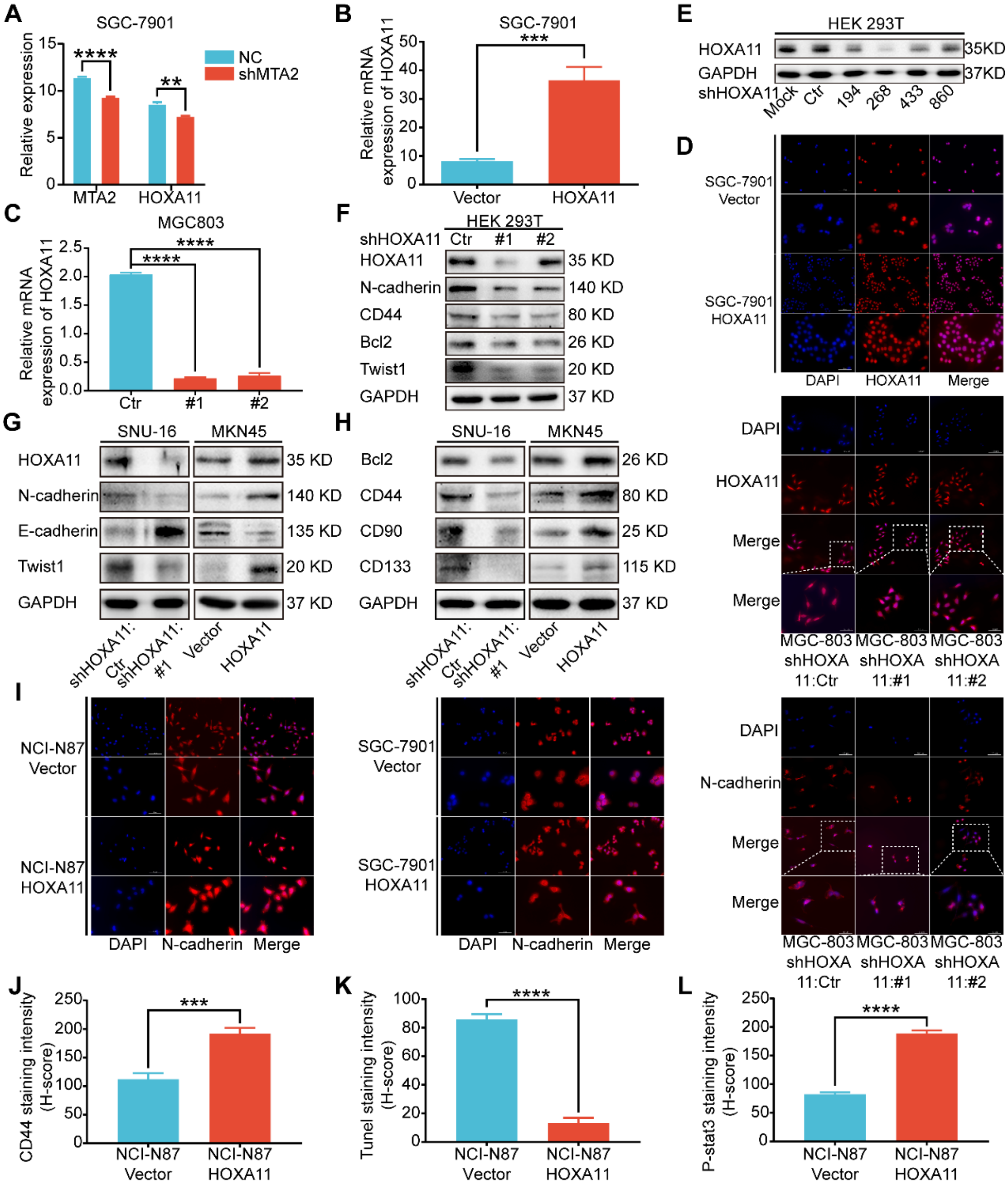

Supplementary figure 2. (A & B) SGC-7901-Vector and SGC-7901-HOXA11 cells had been treated with either BBI608 (6.5 μM) or DMSO for 24 h at 37°C were analyzed using western blot with the indicated antibodies. GAPDH was used as the internal protein loading control. Each experiment was performed in triplicate. (C) The protein expression of Arpc2 and Arpc3 in SGC-7901-Vector and SGC-7901-HOXA11 cells treated with CK636 (4 μM) for 24 h at 37°C were analyzed using western blot with the indicated antibodies. GAPDH was used as the internal protein loading control. Each experiment was performed in triplicate. (D) SGC-7901-HOXA11 cells were treated with knockdown of CD44s for 24 h at 37°C. Cell lysates from the indicated cells were then analyzed by western blot with the indicated antibodies. GAPDH was used as the internal protein loading control. Each experiment was performed in triplicate. (E) SGC-7901-HOXA11 cells were treated with AG490 (60 μM) for 2 h at 37°C. Cell lysates from the indicated cells were then analyzed by western blot with the indicated antibodies. GAPDH was used as the internal protein loading control. Each experiment was performed in triplicate. (F) The indicated cells were grown for 3 days and then treated with BBI608, and viability was assessed after 24 h, each experiment was performed in triplicate. The data (IC50, μM) represent averages of three separate experiments. (G) The map of Stat3 binding sites in the promoter region of HOXA11. (H) Luciferase reporter assay was used for the detection of Stat3 binding sites in the promoter region of HOXA11. (I) luciferase activity of HOXA11 promoter was determined in NCI-N87 and SGC-7901 cells treated with BBI608 or DMSO. Results were shown as mean ± SEM of three independent experiments, each experiment was performed in triplicate. NS, no significance; ****, $P<0.0001$ (Student *t* test).

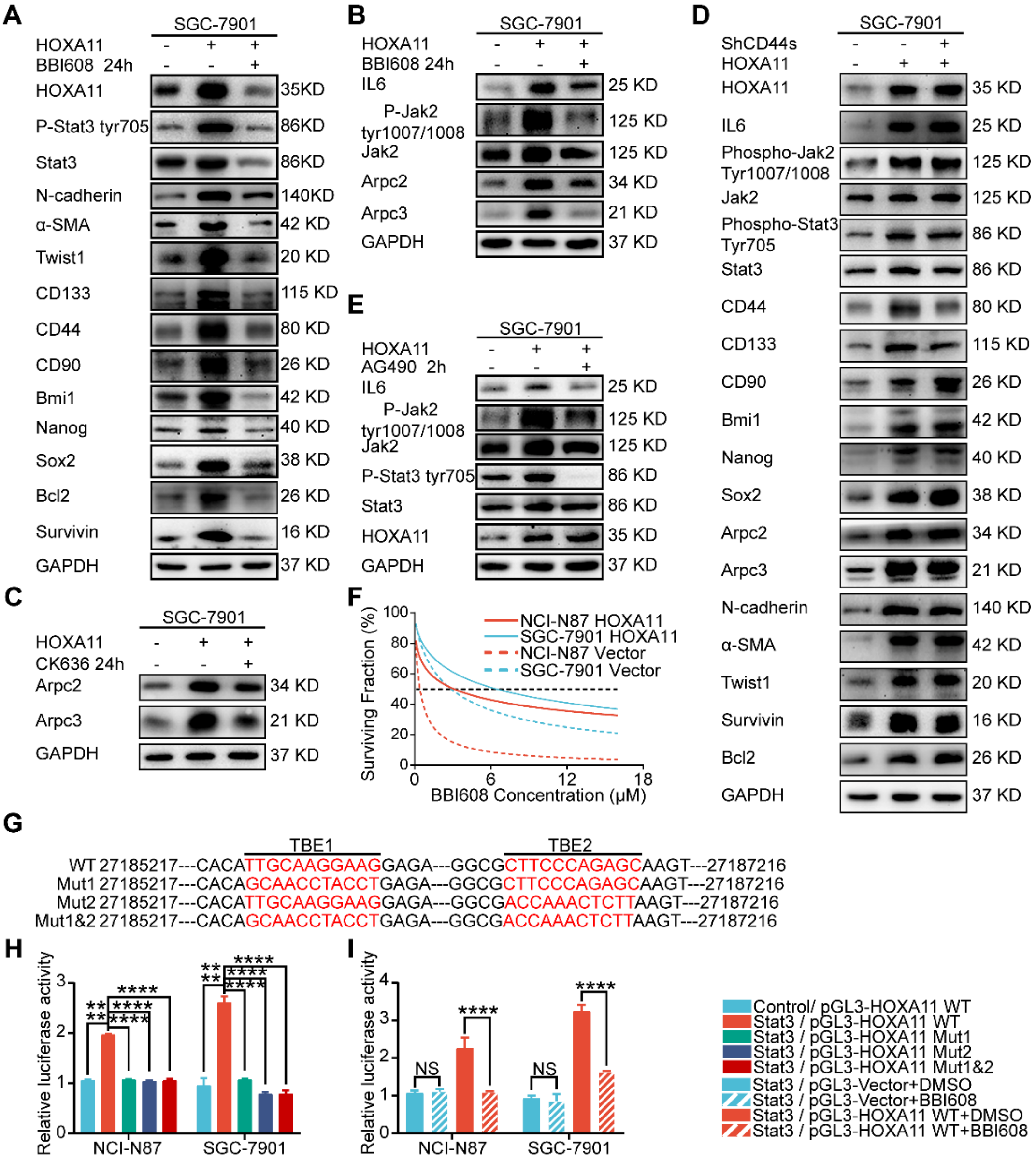
A
SGC-7901
HOXA11 - + +
BBI608 24h - - +
HOXA11 35KD
P-Stat3 tyr705 86KD
Stat3 86KD
N-cadherin 140KD
α-SMA 42 KD
Twist1 20 KD
CD133 115 KD
CD44 80 KD
CD90 26 KD
Bmi1 42 KD
Nanog 40 KD
Sox2 38 KD
Bcl2 26 KD
Survivin 16 KD
GAPDH 37 KD
B
SGC-7901
HOXA11 - + +
BBI608 24h - - +
IL6 25 KD
P-Jak2 tyr1007/1008 125 KD
Jak2 125 KD
Arpc2 34 KD
Arpc3 21 KD
GAPDH 37 KD
C
SGC-7901
HOXA11 - + +
CK636 24h - - +
Arpc2 34 KD
Arpc3 21 KD
GAPDH 37 KD
D
SGC-7901
ShCD44s - - +
HOXA11 - + +
HOXA11 35 KD
IL6 25 KD
Phospho-Jak2 Tyr1007/1008 125 KD
Jak2 125 KD
Phospho-Stat3 Tyr705 86 KD
Stat3 86 KD
CD44 80 KD
CD133 115 KD
CD90 26 KD
Bmi1 42 KD
Nanog 40 KD
Sox2 38 KD
Arpc2 34 KD
Arpc3 21 KD
N-cadherin 140 KD
α-SMA 42 KD
Twist1 20 KD
Survivin 16 KD
Bcl2 26 KD
GAPDH 37 KD
E
SGC-7901
HOXA11 - + +
AG490 2h - - +
IL6 25 KD
P-Jak2 tyr1007/1008 125 KD
Jak2 125 KD
P-Stat3 tyr705 86 KD
Stat3 86 KD
HOXA11 35 KD
GAPDH 37 KD
F
NCI-N87 HOXA11
SGC-7901 HOXA11
NCI-N87 Vector
SGC-7901 Vector
Surviving Fraction (%)
BBI608 Concentration (μM)
G
TBE1
TBE2
WT 27185217---CACATTGCAAGGAAGGAGA---GGCGCTTCCCAGAGCAAGT---27187216
Mut1 27185217---CACAGCAACCTACCTGAGA---GGCGCTTCCCAGAGCAAGT---27187216
Mut2 27185217---CACATTGCAAGGAAGGAGA---GGCGACCAAACTCTTAAGT---27187216
Mut1&2 27185217---CACAGCAACCTACCTGAGA---GGCGACCAAACTCTTAAGT---27187216
H
Relative luciferase activity
NCI-N87
SGC-7901
I
Relative luciferase activity
NS
NCI-N87
SGC-7901
Control/ pGL3-HOXA11 WT
Stat3 / pGL3-HOXA11 WT
Stat3 / pGL3-HOXA11 Mut1
Stat3 / pGL3-HOXA11 Mut2
Stat3 / pGL3-HOXA11 Mut1&2
Stat3 / pGL3-Vector+DMSO
Stat3 / pGL3-Vector+BBI608
Stat3 / pGL3-HOXA11 WT+DMSO
Stat3 / pGL3-HOXA11 WT+BBI608

Supplementary figure 3. (A) BBI608, which targets Stat3, suppressed the peritoneal metastasis of MGC-803-Control cells in BALB/c mice. Tumor in all groups were measured both in situ and after laparotomy. (B) Statistical analysis of the bioluminescence in peritoneal foci of all groups. Results were shown as mean ± SEM, ****, P<0.0001 (the analysis of variance test). (C) These mice were given BBI608 (20 mg/kg), or DMSO i.p. All regimens were administered twice a week. Body weight was measured weekly during the treatment. There was no significant decrease in body weight due to administration of the BBI608. (D) Immunohistochemistry assay shown hematoxylin & eosin (H & E) and the expression of HBME-1 and Calretitin in peritoneal foci derived from NCI-N87-Vector, NCI-N87-HOXA11, SGC-7901-Vector and SGC-7901-HOXA11 cell groups. The scale bar, from left to right, 400 μm, 100× magnification; 200 μm, 200× magnification; 100 μm, 400× magnification. Each experiment was performed in triplicate. Arrow point to the area: T, tumor; M, mesothelium. (E & F) Statistical analysis of HBME-1 and Calretitin staining intensity (H-score) in both groups. Results were shown as mean ± SEM of three independent experiments, each experiment was performed in triplicate. ***, P<0.001; ****, P<0.0001 (Student *t* test).

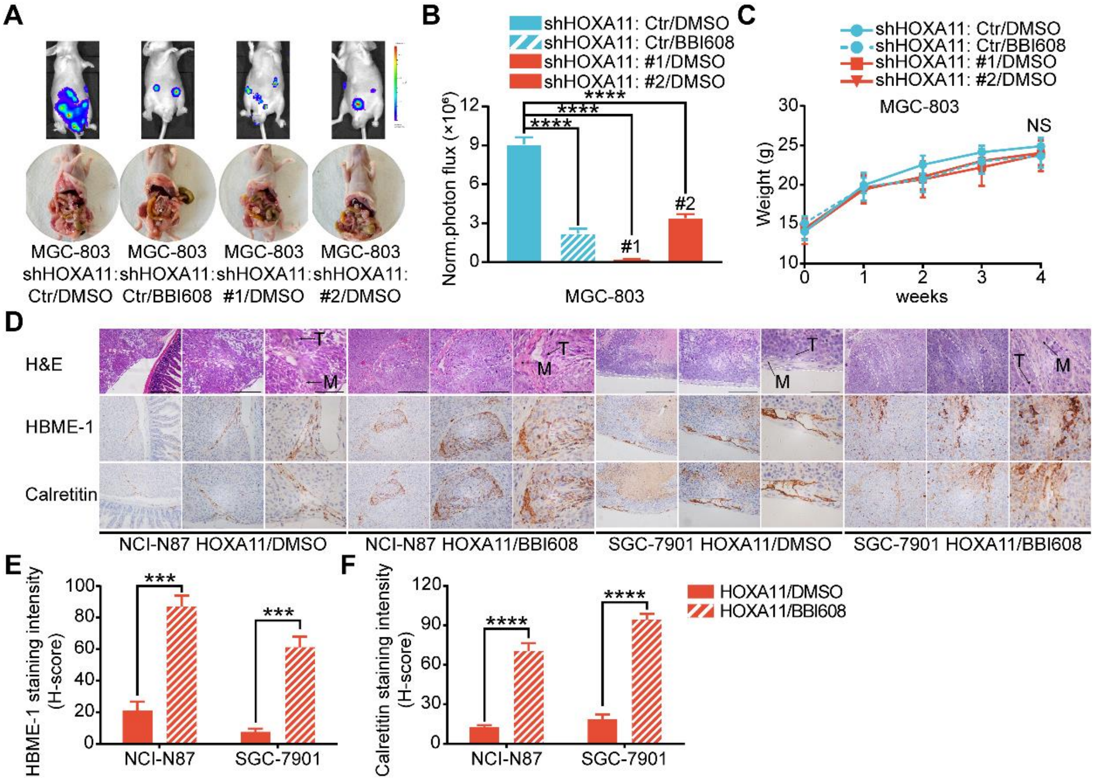
A
MGC-803 shHOXA11: Ctr/DMSO
MGC-803 shHOXA11: Ctr/BBI608
MGC-803 shHOXA11: #1/DMSO
MGC-803 shHOXA11: #2/DMSO
B
shHOXA11: Ctr/DMSO
shHOXA11: Ctr/BBI608
shHOXA11: #1/DMSO
shHOXA11: #2/DMSO
Norm.photon flux (×10⁶)
****
#1
#2
MGC-803
C
shHOXA11: Ctr/DMSO
shHOXA11: Ctr/BBI608
shHOXA11: #1/DMSO
shHOXA11: #2/DMSO
MGC-803
NS
Weight (g)
weeks
D
H&E
HBME-1
Calretitin
T
M
NCI-N87 HOXA11/DMSO
NCI-N87 HOXA11/BBI608
SGC-7901 HOXA11/DMSO
SGC-7901 HOXA11/BBI608
E
HBME-1 staining intensity (H-score)
***
NCI-N87
SGC-7901
F
Calretitin staining intensity (H-score)
****
HOXA11/DMSO
HOXA11/BBI608

Supplementary figure 4. (A) Statistical analysis of the HOXA11 expression in the paired primary GC and peritumor tissues of validation cohort by MOD of staining. ****, P<0.0001 (Student *t* test). (B) Statistical analysis of the HOXA11 expression in peritumor tissues of AJCC stage I and primary GC tissues of different AJCC stages in validation cohort by MOD of staining. NS, no significance; ****, P<0.0001 (the analysis of variance test). (C to E) The relative mRNA expression of HOXA11 in GSE13861 (peritumor=19, tumor=65), GSE13911 (peritumor=30, tumor=30) and TCGA cohort (peritumor=32, tumor=375). ****, P<0.0001 (Student *t* test). (F) Survival of patients in HOXA11-low expression group and HOXA11-high expression group. The survival time of patients in the validation cohort was compared between groups using the Mantel-Cox test, which presented significantly longer survival of patients in the HOXA11-low expression group (P<0.0001). (G) GC patients (GEO database from Kaplan-Meier plotter) with high HOXA11 expression (n=652) have lower OS rates than patients with low HOXA11 expression (n=224) do (P<0.0001). (H) Univariate analysis was performed in validation cohort. The bar correspond to 95% confidence intervals. (I) Multivariate analysis was performed in validation cohort. The bar correspond to 95% confidence intervals.

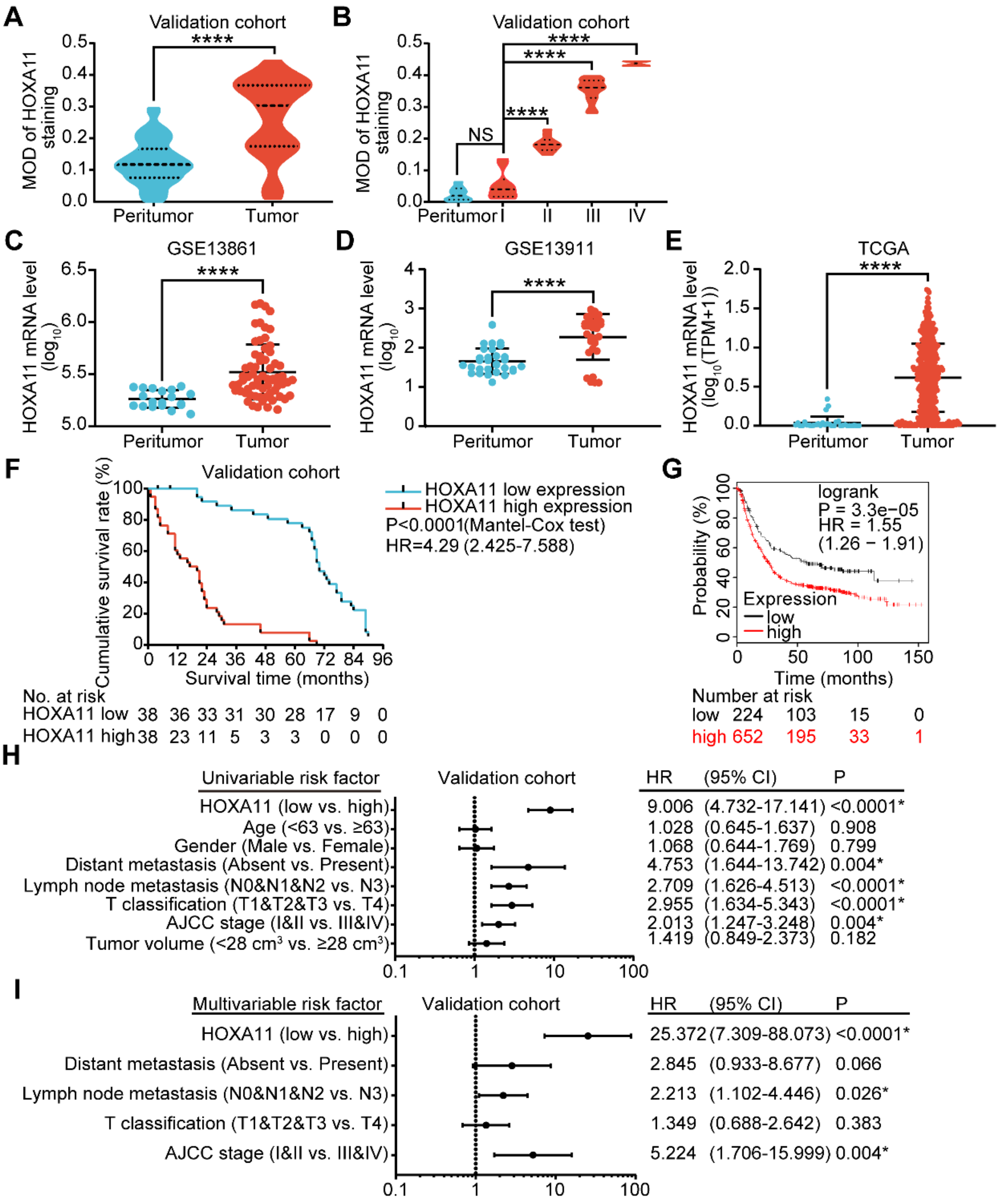
A
Validation cohort
MOD of HOXA11 staining
****
Peritumor
Tumor
B
Validation cohort
MOD of HOXA11 staining
NS
****
Peritumor
I
II
III
IV
C
GSE13861
HOXA11 mRNA level (log10)
****
Peritumor
Tumor
D
GSE13911
HOXA11 mRNA level (log10)
****
Peritumor
Tumor
E
TCGA
HOXA11 mRNA level (log10(TPM+1))
****
Peritumor
Tumor
F
Validation cohort
Cumulative survival rate (%)
Survival time (months)
HOXA11 low expression
HOXA11 high expression
P<0.0001(Mantel-Cox test)
HR=4.29 (2.425-7.588)
No. at risk
HOXA11 low 38 36 33 31 30 28 17 9 0
HOXA11 high 38 23 11 5 3 3 0 0 0
G
logrank
P = 3.3e−05
HR = 1.55
(1.26 − 1.91)
Probability (%)
Expression
low
high
Time (months)
Number at risk
low 224 103 15 0
high 652 195 33 1
H
Univariable risk factor
Validation cohort
HR (95% CI) P
HOXA11 (low vs. high) 9.006 (4.732-17.141) <0.0001*
Age (<63 vs. ≥63) 1.028 (0.645-1.637) 0.908
Gender (Male vs. Female) 1.068 (0.644-1.769) 0.799
Distant metastasis (Absent vs. Present) 4.753 (1.644-13.742) 0.004*
Lymph node metastasis (N0&N1&N2 vs. N3) 2.709 (1.626-4.513) <0.0001*
T classification (T1&T2&T3 vs. T4) 2.955 (1.634-5.343) <0.0001*
AJCC stage (I&II vs. III&IV) 2.013 (1.247-3.248) 0.004*
Tumor volume (<28 cm³ vs. ≥28 cm³) 1.419 (0.849-2.373) 0.182
I
Multivariable risk factor
Validation cohort
HR (95% CI) P
HOXA11 (low vs. high) 25.372 (7.309-88.073) <0.0001*
Distant metastasis (Absent vs. Present) 2.845 (0.933-8.677) 0.066
Lymph node metastasis (N0&N1&N2 vs. N3) 2.213 (1.102-4.446) 0.026*
T classification (T1&T2&T3 vs. T4) 1.349 (0.688-2.642) 0.383
AJCC stage (I&II vs. III&IV) 5.224 (1.706-15.999) 0.004*

Supplementary figure 5. Nomogram predicting 3- and 5-year overall survival for GC. (A) The nomogram was used by sum of the points identified on the points scale for each variant. The total points projected on the bottom scales mean the probability of 3- and 5-year survival. (B & C) The calibration curve for predicting OS at 3- and 5-years in training cohort. (D & E) The calibration curve for predicting OS at 3- and 5-years in validation cohort.

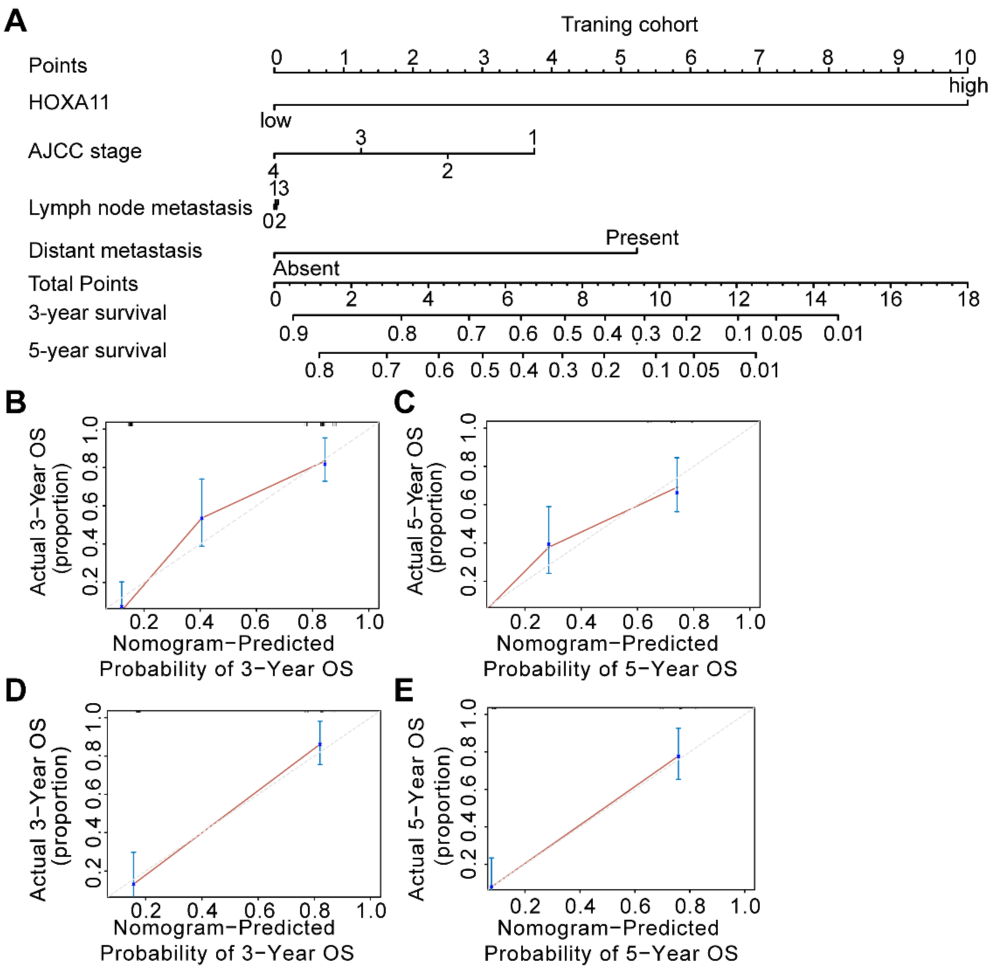

Supplementary table 1 clinicopathological characteristics of patient samples and expression of HOXA11 in training and validation cohort

Training cohort Characteristics	Number of cases (%)	Validation cohort Characteristics	Number of cases (%)
HOXA11		HOXA11	
Low	56 (49.1)	Low	38 (50)
High	58 (50.9)	High	38 (50)
AJCC Stage		AJCC Stage	
I&II	47 (41.2)	I&II	32 (42.1)
III&IV	67 (58.8)	III&IV	44 (57.9)
T phase		T phase	
1/2/3	89 (78.1)	1/2/3	60 (78.9)
4	25 (21.9)	4	16 (21.1)
Lymph node metastasis		Lymph node metastasis	
0/1/2	72 (63.2)	0/1/2	50 (65.8)
3	42 (36.8)	3	26 (34.2)
Gender		Gender	
Male	63 (55.3)	Male	54 (71.1)
Female	51 (44.7)	Female	22 (28.9)
Age		Age	
<63	50 (43.9)	<63	39 (51.3)
≥63	64 (56.1)	≥63	37 (48.7)
Distant metastasis		Distant metastasis	
Yes	8 (7)	Yes	4 (5.3)
No	106 (93)	No	72 (94.7)
Tumor volume		Tumor volume	
<27.8	85 (74.6)	<27.8	53 (69.7)
≥27.8	29 (25.4)	≥27.8	23 (30.3)

Supplementary table 2 Correlations between HOXA11 expression and clinical characteristics in Training cohort (n=114)

Clinicopathologic parameters		expression Low	High	Total	P value
Distant metastasis	No	56	50	106	
	Yes	0	8	8	
		56	58	114	0.006
Gender	Male	35	28	63	
	Female	21	30	51	
		56	58	114	0.127
Age	<63	26	24	50	

	≥63	30	34	64	
		56	58	114	0.587
AJCC stage	I/II	47	0	47	
	III/IV	9	58	67	
		56	58	114	<0.0001
T phase	T1/T2/T3	53	36	89	
	T4	3	22	25	
		56	58	114	<0.0001
Lymph node metastasis	N0/N1/N2	50	22	72	
	N3	6	36	42	
		56	58	114	<0.0001
Tumor volume	<28 cm3	47	38	85	
	≥28 cm3	9	20	29	
		56	58	114	0.024

Supplementary table 3 Correlations between HOXA11 expression and clinical characteristics in validation cohort (n=76)

Clinicopathologic parameters		expression		Total	P value
		Low	High		
Distant metastasis	No	38	34	72	
	Yes	0	4	4	
		38	38	76	0.115
Gender	Male	28	26	54	
	Female	10	12	22	
		38	38	76	0.801
Age	<63	19	20	39	
	≥63	19	18	37	
		38	38	76	0.818
AJCC stage	I/II	32	0	32	
	III/IV	6	38	44	
		38	38	76	<0.0001
T phase	T1/T2/T3	37	23	60	
	T4	1	15	16	
		38	38	76	<0.0001
Lymph	N0/N1/N2	36	14	50	

node metastasis					
	N3	2	24	26	
		38	38	76	<0.0001
Tumor volume	<28 cm3	32	21	53	
	≥28 cm3	6	17	23	
		38	38	76	0.006

Supplementary table 4 Antibodies, source and dilution

Antibody	Source	Dilution (application)
HOXA11	Abcam; #AB54365	1:1000(WB)<br>1:250 (IHC)<br>1:500 (IF)
CD44	Cell Signaling Technology; #3570	1:1000(WB)<br>1:400(IF)
CD44	Proteintech Group; #15675-1-AP	1:500(IHC)
Survivin	Cell Signaling Technology; #2808	1:1000(WB)
Stat3	Cell Signaling Technology; #4904T	1:1000(WB)<br>1:50(ChIP)
TWIST1	Abclonal; #A3237	1:1000(WB)<br>1:100(IHC)
Jak2	Cell Signaling Technology; #3230S	1:1000(WB)
Phosphor-Jak2(Tyr1007/1008)	Cell Signaling Technology; #3771S	1:1000(WB)
N-cadherin	Cell Signaling Technology; #14215S	1:1000(WB)<br>1:200(IF)
E-cadherin	Proteintech Group; #20874-1-AP	1:1000(WB)
Bmi1	Abclonal; #A0211	1:1000(WB)
Nanog	Abclonal; #A14150	1:1000(WB)
Sox2	Abclonal; #A11501	1:1000(WB)
Fibronectin	Abclonal; #A12932	1:1000(WB)
IL6	Abcam; #AB154367	1:1000(WB)
HBME-1	DAKO; #M3505	1:50(IHC)
Calretinin	DAKO; #7245	1:50(IHC)
Tunel	Calbiochem; #QIA33	
Phosphor-Stat3(Tyr705)	Cell Signaling Technology; #9145	1:1000(WB)<br>1:100(IHC)
Arpc2	Abclonal; #A10791	1:1000(WB)
Arpc3	Abclonal; #A7767	1:1000(WB)

CD133	Abclonal; #A12711	1:1000(WB)
α-SMA	Abcam; #AB5694	1:1000(WB)
Bcl2	Cell Signaling Technology; #15071S	1:1000(WB)
CD90	Santa Cruz; #SC-19614	1:1000(WB)
Cy3-Mouse	Proteintech Group; # SA00009-1	1:100(IF)
Cy3-Rabbit	Proteintech Group; # SA00009-2	1:100(IF)
GAPDH-Mouse	Cell Signaling Technology; #51332	1:1000(WB)
GAPDH-Rabbit	Cell Signaling Technology; #5174	1:1000(WB)
Histone H3	Cell Signaling Technology; #4499	1:1000(WB)
DAPI	Beyotime; #C1002	1:1000(IF)
TRITC Phalloidin	Yeasen; #40734ES75	1:100(IF)
Flag	Abclonal; #AE005	1:1000(WB)
Bax	Cell Signaling Technology; #5023s	1:1000(WB)
PARP/Cleaved PARP	Cell Signaling Technology; #9532s	1:1000(WB)
Caspase3/Cleaved Caspase3	Cell Signaling Technology; #9662s	1:1000(WB)

Supplementary table 5 Primer sequences

Primer	Forward	Reverse
GAPDH	GGACCTGACCTGCCGTCTAG	GTAGCCCAGGATGCCCTTGA
HOXA11	TGCCAAGTTGTACTTACTACGTC	GTTGGAGGAGTAGGAGTATGTCA
HOXA11-ChIP-1	TGAGCAGTAGGAAAGGCACAGA	GGGAGAATGGAGGTGGAAAGGA
HOXA11-ChIP-2	GCAAATCGGACAAGCCACCA	CCATAGACTTGCTCTGGGAAGC

Supplementary table 6 The sequence of targeting genes by shRNA-mediated knockdown

Gene’s name	Sequence
Sh Negative Control	TTCTCCGAACGTGTCACGT
shHOXA11-194	GCCCAATGACATACTCCTACT
shHOXA11-268	GCCATTGAGCCCGCCACTAAA
shHOXA11-433	GCAGTCTCGTCCAATTTCTAT
shHOXA11-860	GCGTCTACATTAACAAAGAGA
shCD44s	GTCAACAGTCGAAGAAGGTGT
